# Gelatin–Tannin-Based Greener Binder Technology for Stone Shot and Stone Wool Materials: A Detailed Study

**DOI:** 10.1021/acsomega.1c05153

**Published:** 2021-11-30

**Authors:** Thomas Hjelmgaard, Josefine Øgaard Svendsen, Berthold Köhler, Paul Pawelzyk, Dorthe Lybye, Carina Michella Schmücker, Peter Reiter, Matthias Reihmann, Peter Anker Thorsen

**Affiliations:** † ROCKWOOL International A/S, Hovedgaden 584, Entrance C, 2640 Hedehusene, Denmark; ‡ GELITA AG, Uferstrasse 7, 69502 Eberbach, Germany

## Abstract

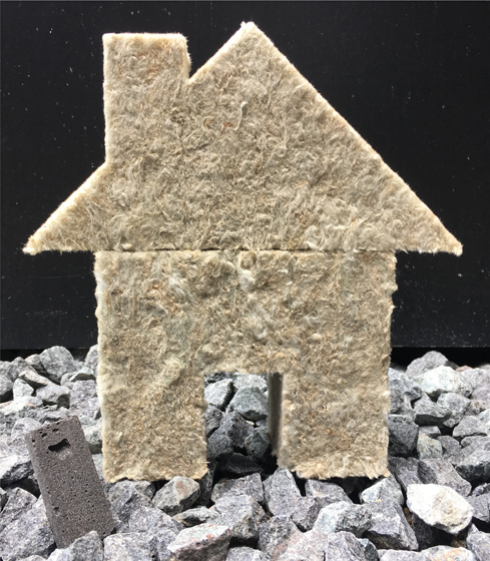

The detailed study presented herein of gelatins modified with tannins as greener binder systems for stone wool and related materials has unveiled a versatile technology that offers a wide range of possibilities for tailor-making properties toward various application areas. Thus, high unaged and aged mechanical strengths in combination with low water solubilities may generally be obtained from the use of gelatins with higher gel strength (and hence, generally, higher molecular weights), low-to-mid range tannin addition levels (3–20%), alkali metal hydroxides for pH adjustment, and final pH in the range 8–9. Comparatively low water uptake properties may be obtained using higher gel strength type A gelatins, lower tannin addition levels, alkali metal hydroxides for pH adjustment, and lower final pH. Even lower water uptake properties may then be obtained using Ca(OH)_2_ in place of alkali metal hydroxides. If desired, higher water uptakes may be obtained using type B gelatins (or lower gel strength gelatins in general), higher tannin addition levels, and higher final pH. Mechanistic studies indicated that the optimal modification of gelatin with the tannin component occurs via several pathways.

## Introduction

Stone wool is a remarkably versatile material, which is finding an increasing use in addressing critical global challenges. Stone wool products, for example, significantly improve the energy efficiency of buildings, protect against fire, and improve acoustic comfort. Furthermore, stone wool materials can also be used in water management systems to counter the effects of climate changes and in precision growing applications to improve the efficiency of fresh food production. A stone wool product is—at least in its very basic form—a conceivably simple material composed of stone wool fibers glued together by an adhesive, termed a binder (typically present in an amount of 1–10 wt % of the fibers). Stone wool is conventionally produced by spraying an aqueous binder system onto stone wool fibers formed from a stone melt in a cascade spinning process.^1,2^ The resulting web is collected and transferred into a curing oven, where the binder is cured by blowing heated air through the stone wool.^1,2^ Stone wool binder systems are traditionally based on phenol–formaldehyde and phenol–urea–formaldehyde resins.^3,4^ Other examples include acrylic-based and carbohydrate-based binders. Although strong, the conventional binder systems typically include components that are harmful in uncured form. In addition, they generally require high temperatures to cure properly (*i.e*., well above 200 °C), leading to high energy consumption and possibly also emissions that may require post-treatment.

Striving to address these sustainability problems, the invention of greener binder systems based on nontoxic biopolymers, which were able to cure in the vicinity of ambient temperatures, was recently disclosed.^5−9^ Accordingly, the first systematic studies and direct comparisons of various properties of binder systems based on type A gelatins modified with tannin from chestnut trees in the presence of NaOH at pH 9 or with transglutaminase at pH 5 were recently published.^10^ As an essential element in advancing and understanding the greener binder technology based on gelatins modified with tannins, this paper presents the first systematic studies of the effects of varying the nature of the gelatin component, the tannin component, the metal cation of the hydroxide base, and the final pH. Apart from unaged and aged mechanical strengths and binder solubilities obtained from the use of a convenient composite bar model method, the first systematic water uptake studies of all of the binder compositions and comparisons with contact angles of selected series are likewise reported. Importantly, studies of the mechanism of the modification of gelatin with tannin when in contact with stone shots are also presented for the first time.

### Gelatins and Tannins as Binder Components

**Figure 1:**
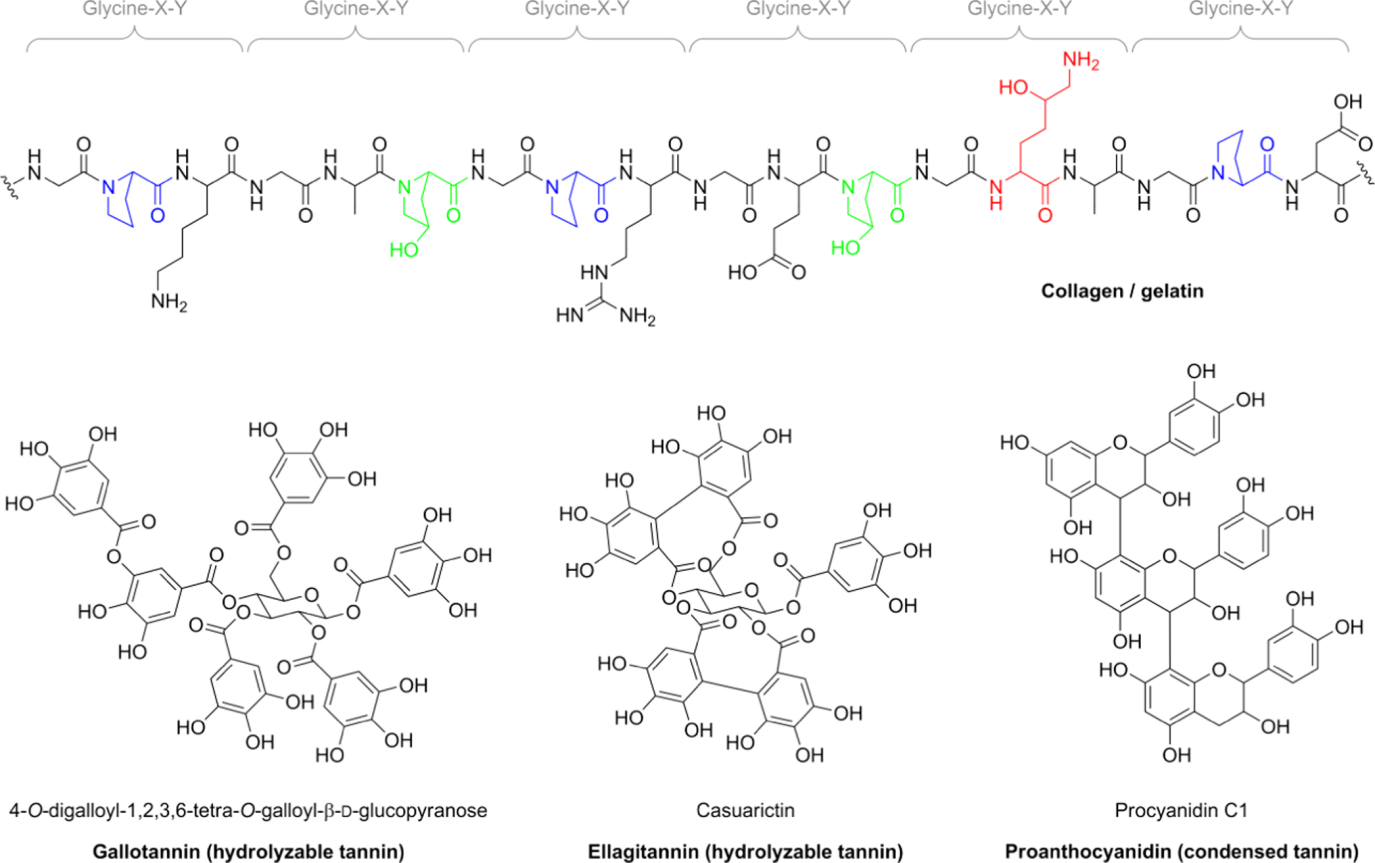
Top: Schematic illustration of the glycine-X-Y sequence of collagen and gelatin. Proline, hydroxyproline, and hydroxylysine residues are highlighted in blue, green, and red, respectively. Bottom: Examples of gallotannin (hydrolyzable tannin, left), ellagitannin (hydrolyzable tannin, mid), and proanthocyanidin (condensed tannin, right).

Gelatin is a natural protein conventionally made by acidic or basic treatment of animal-derived leftovers that contain collagen.^11,12^ While the amino acid compositions of native collagen and the acid-processed type A gelatins are quite similar, both asparagine and glutamine residues are almost completely hydrolyzed to aspartic and glutamic acid residues, respectively, in the base-processed type B gelatins.^11^ Collagen and the derived gelatins generally comprise repetitive units of the general sequence glycine-X-Y (Figure 1, top). A rather unique property of collagen and gelatin is the high content of the amino acids proline (∼12%) and hydroxyproline (∼10%) as well as the presence of hydroxylysine (∼0.5%).^11,12^ Proline and hydroxyproline limit the rotation about the amino acid backbone, thereby contributing to the stabilization of the unique triple helix structure of collagen.^11,12^ In the case of gelatin, a partial renaturation at lower temperatures results in the well-known formation of thermoreversible gels with a gelling power in the range of 50–300 bloom. Gelatin molecules range from 5 to 800 kDa in size, and high gelling power gelatins will be characterized to some extent by high average molecular weights, while low gelling power gelatins normally are characterized by low average molecular weights.^11^

In addition to warm water solubility and gel formation, gelatin is able to form strong films and displays adhesive properties.^10−14^ However, it is also often desirable (though not always necessary) that the final dried and cured binder system is able to withstand humid or wet conditions. To that end, many phenolics^15^ such as the structurally immensely diverse tannins^16−19^ are well known for their capacity to interact strongly with proteins such as gelatin.^10,12,16,17,20−29^ One important class of tannins is the hydrolyzable tannins, which comprise gallotannins and ellagitannins (Figure 1, bottom left and mid, respectively).^16−18^ Another important class of tannins consists of the condensed tannins or proanthocyanidins (Figure 1, bottom right).^16−19^ Two pathways leading to strong interactions between tannins and proline-rich proteins such as gelatins are prevalent in the literature. The first pathway is based on a complex combination of noncovalent interactions^16,20−25^ and may be explained by hydrophobic stacking of the phenolic rings against the proline rings (σ–π attraction) and formation of hydrogen bonds between the phenolic hydroxy groups and the carbonyl groups linked to the proline amino groups.^16^ The second pathway occurs by the formation of covalent bonds between the tannins and gelatins under oxidative conditions.^16,22,26^ The reaction sequence is promoted by basic conditions, where phenolate anions derived from the tannins may readily be oxidized to yield electrophilic quinone species, which then react with nucleophiles such as the lysine side chains from gelatins.

## Results and Discussion

The binder studies presented herein were performed using an improved version of a convenient and versatile model method^10^ based on the manufacture and testing of composite bars produced from 15 wt % aqueous binder solutions and submillimeter diameter stone shots^2^ obtained from the production of stone wool fibers. The bars were cured according to the requirement of the binder system under investigation (≥200 °C/1 h for conventional binder systems; rt/2–4 days for the binders presented herein) and had final binder contents of approximately 2.9%. The ensuing treatments include accelerated aging by autoclave treatment (15 min/120 °C/1.2 bar) or water bath treatment (3 h/80 °C), measurement of unaged and aged mechanical strengths in a three-point bending test, and measurement of binder contents (590 °C/30 min). A value for binder solubility was calculated as the difference between unaged and water bath-aged binder contents. This expedient model method is capable of predicting the relative strengths and properties of binders remarkably well across highly varied binder systems. Bars produced using conventional binder systems display unaged and aged mechanical strengths in the range of 0.25–0.40 kN and 0.15–0.30 kN, respectively, and thus typically lose 30–50% of their initial strength during the accelerated aging treatment.^30^ In addition, it has now been found that the model method may also be used to predict the relative water uptake properties within the binder systems. The measurement of this essential parameter was achieved by submerging the unaged composite bars in water for 3 and 24 h at room temperature and measuring the weight increase.

### Variations in Gelatins

In the preceding study, it was shown that increased unaged and aged mechanical strengths combined with decreased binder solubilities were generally observed as a function of increased gel strength and viscosity of the gelatin component.^10^ Modification of gelatin was carried out at pH 9 in the presence of NaOH, where the inclusion of low-to-mid amounts of tannin from chestnut trees (TC) appeared to be optimal (3–20%, calculated on the basis of the gelatin component). These observations were made using the four type A gelatins: GA78, GA120, GA180, and GA305 (where the letter A designates a type A gelatin and the number represents the bloom strength). Apart from increasing gel strengths, these gelatins are also characterized by increasing viscosity in solution (see the SI for details). To investigate the impact of the viscosity parameter, a further comparative study was carried out herein using the high-viscosity type A gelatin GA291v (see the SI for details). While only minor effects were observed on the unaged strengths when compared to GA305, the increase in viscosity of GA291v generally appeared to have a positive effect on the water bath-aged strengths, while the autoclave-aged strengths generally decreased. The viscosity parameter should therefore be considered when developing these gelatin-based binder systems.

**Figure 2:**
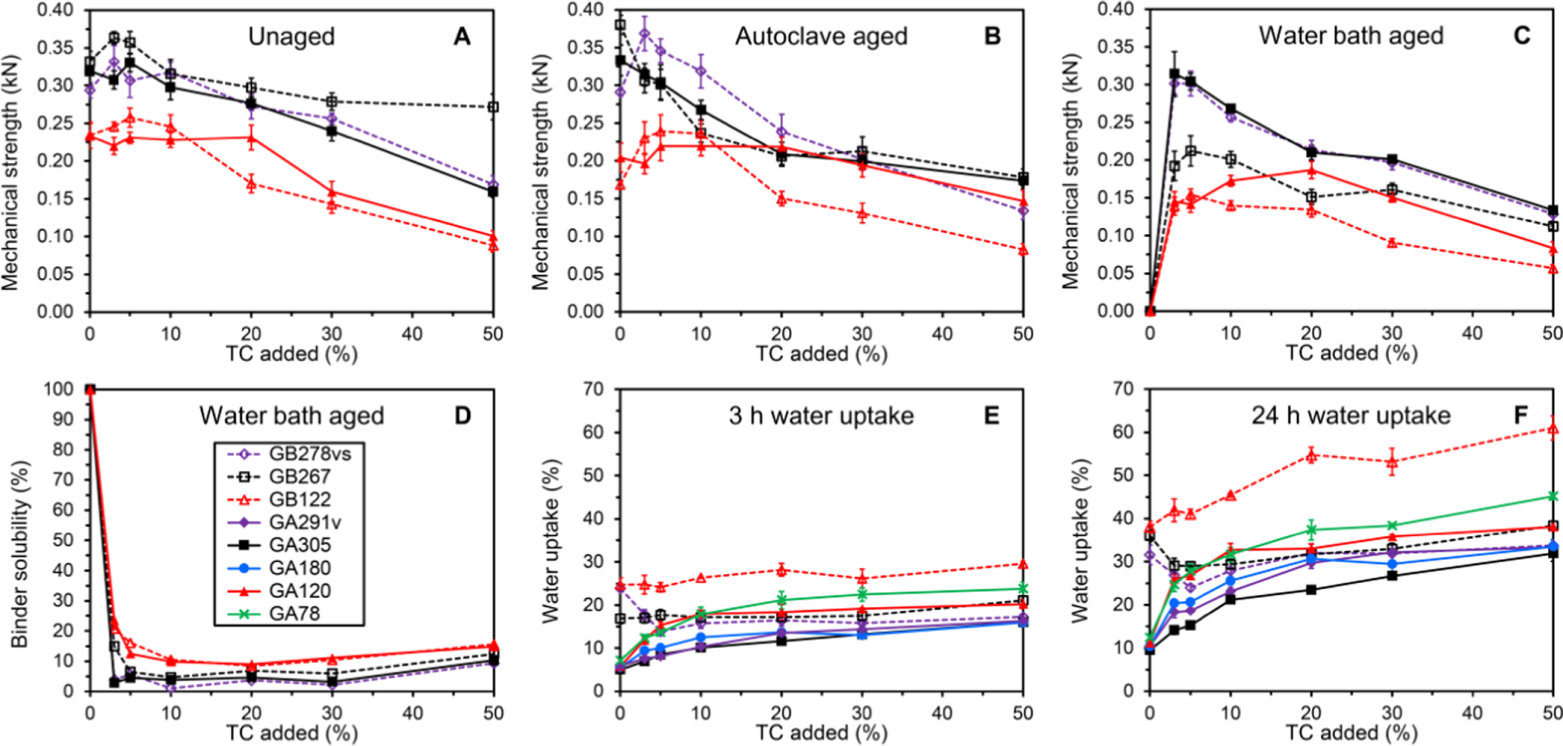
Overview of unaged and aged mechanical strengths (A–C), binder solubility (D), and water uptake properties (E, F) of composite bars made from stone shots and GB278vs (◊, violet, dashed lines), GB267 (□, black, dashed lines), GB122 (Δ, red, dashed lines), GA291v (⧫, violet), GA305 (■, black), GA180 (●, blue), GA120 (▲, red), and GA78 (×, green) modified with TC in the presence of NaOH at pH 9. Data for the mechanical strengths and water uptakes are expressed as mean ± standard error (*n* = 5 and *n* = 3, respectively).

The water uptakes measured after 3 and 24 h for bars made with all five type A gelatins GA78, GA120, GA180, GA305, and GA291v modified with 0–50% TC in the presence of NaOH at pH 9 are illustrated in Figure 2E,F. The water uptakes generally increased with increasing tannin addition. The most significant increases were observed in the lower tannin addition range (0–10%), where the water uptake more than doubled. Contact angle measurements made on films produced from GA120 modified with 0–50% TC in the presence of NaOH at pH 9 were in accordance with these general findings (see the SI for details). In addition, the water uptake generally increased with decreasing gel strength of the gelatin component (Figure 2E,F). Overall, these results indicate that high water uptake is favored by the inclusion of higher amounts of tannin combined with low average molecular weights of the gelatin component. Conversely, low water uptake characteristics may be obtained by a combination of low tannin content and high average molecular weights of the gelatin.

The effect of using type B gelatins in place of type A gelatins was investigated using GB122, GB267, and GB278vs as model gelatins. These type B gelatins are likewise characterized by increasing gel strengths and viscosity (see the SI for details). GB278vs is a low-salt grade gelatin with low conductivity, which is characterized by a considerably higher viscosity in solution than GB267 (see the SI for details). An overview of the results obtained for bars produced with GB122, GB267, and GB278vs modified with 0–50% TC in the presence of NaOH at pH 9 is shown in Figure 2. Mechanical strengths and binder solubilities previously disclosed for type A gelatins GA120 and GA305 are also included for comparison.^10^ Analogous to the observations previously made for type A gelatins, the two high strength type B gelatins GB267 and GB278vs generally produced bars that were stronger both before and after aging than the bars made with the lower strength type B gelatin GB122 (Figure 2A–C). The lower gel strength GB122 generally resulted in bars that were comparable or slightly higher in unaged and aged strengths than the counterpart type A gelatin GA120 at lower tannin contents (up to 10%, Figure 2A–C). At higher tannin contents, GA120 generally performed better than GB122. Interestingly, the two type B gelatins GB267 and GB278vs with comparable high gel strengths but different viscosities and conductivities produced different strength profiles (Figure 2A–C). The most notable difference was the significantly lower water bath-aged strengths obtained from the use of GB267 when compared to GB278vs and GA305 (Figure 2C). The combination of weaker aged strengths and generally slightly higher binder solubility observed for GB267 (Figure 2D) indicates a less efficient modification of this particular gelatin with TC. The observed differences in the strength patterns of GB267 and GB278vs again demonstrate that other gelatin characteristics than gel strength should be observed closely.

Interestingly, the water uptake for bars made from type B gelatins was considerably higher than for bars made from their type A counterparts (Figure 2E,F). This may be the result of the higher content of carboxylic acids in type B gelatins compared to the type A gelatins. These general findings were likewise supported by contact angle measurements made on films produced from GB122 modified with 0–50% TC in the presence of NaOH at pH 9 (see the SI for details). The difference in water uptake was most pronounced for low tannin contents (0–5%, Figure 2E,F). Thus, opposite to type A gelatins, the water uptake was unchanged or even decreased with increasing tannin content in this low tannin range. For >5% tannin, the water uptake then again generally increased with increasing tannin content. A low water uptake is desirable in many stone wool application areas but a higher water uptake may be necessary in application areas such as precision growing or water management applications. These results suggest that changing between type A and type B gelatins may represent one possibility for tailor making the gelatin-based binder systems for such varied application areas.

An important effect of increasing the strength and viscosity of the gelatins is that the setting time of the resulting binder system decreases accordingly. While a short setting time may be useful in some applications, a longer setting time may be desirable in others. To this end, adding a small amount of high strength gelatins to lower strength gelatins may result in significant strength effects while the setting time remains unchanged. Comparative studies of GA120/GA305 90:10 and GA120/GB278vs 90:10 modified with 0–20% TC in the presence of NaOH at pH 9 were therefore carried out (see the SI for details). As desired, no apparent changes to the binder setting time during the manufacture of the bars were observed compared to the use of GA120 as the sole gelatin component. Some significant strength improvements were observed, particularly, in water bath-aged strengths for GA120/GB278vs 90:10. However, the arguably most significant general impact was that the water solubilities decreased to levels closer to those obtained for the use of the higher strength gelatins only. These results demonstrated that significant effects on the properties of gelatin-based binder systems may be obtained by mixing gelatins with different characteristics such as the gel strength and type.

### Variations in Tannins

**Figure 3:**
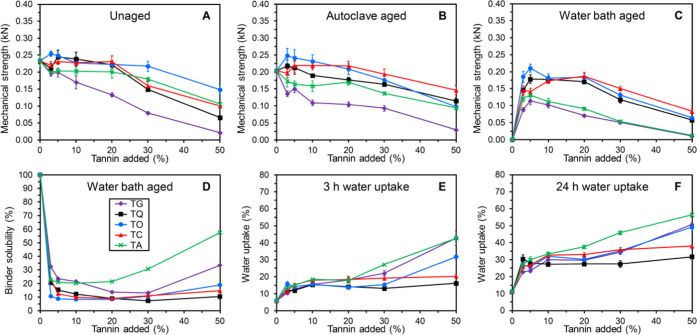
Overview of unaged and aged mechanical strengths (A–C), binder solubility (D), and water uptake properties (E, F) of composite bars made from stone shots and GA120 modified with TG (⧫, violet), TQ (■, black), TO (●, blue), TC (▲, red), and TA (×, green) in the presence of NaOH at pH 9. Data for the mechanical strengths and water uptake are expressed as mean ± standard error (*n* = 5 and *n* = 3, respectively).

The structural diversity of tannins is immense and it can therefore be anticipated that varying the nature of the tannin component will have a major impact on the properties of the resulting binder system. In addition to the previously studied tannin from chestnut trees (TC), tannin from oak trees (TO) and tannic acid (TA) were selected as model hydrolyzable tannins for this study. Tannin from quebracho trees (TQ) and tannin from grape (TG) were selected as representative condensed tannins. An overview of the results obtained for bars produced with GA120 modified with 0–50% TG, TQ, TO, TC, and TA in the presence of NaOH at pH 9 is shown in Figure 3. The mechanical strengths and binder solubilities for modification with TC were reported previously^10^ and are included for comparison.

The highest mechanical strengths in combination with the lowest water solubilities were obtained for modification with low addition levels of TO (3–5%, Figure 3A–D), achieving unaged and aged mechanical strengths that were within the range of conventional binder systems even with the lower strength GA120 as the sole gelatin component. Indeed, the losses in mechanical strength from the aging treatments were less than 15% for the binder composition modified with 5% TO. Overall, TO, TC, and TQ generally provided the highest mechanical strengths in combination with the lowest water solubilities and water uptakes (Figure 3A–F). Especially, the water bath-aged strengths were significantly higher than those obtained with the use of TA and TG (Figure 3C). Accordingly, the water solubilities were also generally significantly higher for the binder compositions comprising TA and TG (Figure 3D). At higher tannin addition levels (≥20%), the use of TA and TG furthermore resulted in comparatively higher water uptake (Figure 3E,F). This combination of results indicated that the modification of gelatin was considerably more efficient with TO, TC, and TQ than with TA and TG. The results clearly demonstrated the large impact the nature of the tannin component has on the properties of the resulting binder system. The selected tannins were of natural sources and hence not completely well defined in their structures, which hampered the establishment of reliable structure–activity relationships. It would therefore be of high interest to complement the present work with studies of well-defined model tannins.

### Variations in Metal Cations

**Figure 4:**
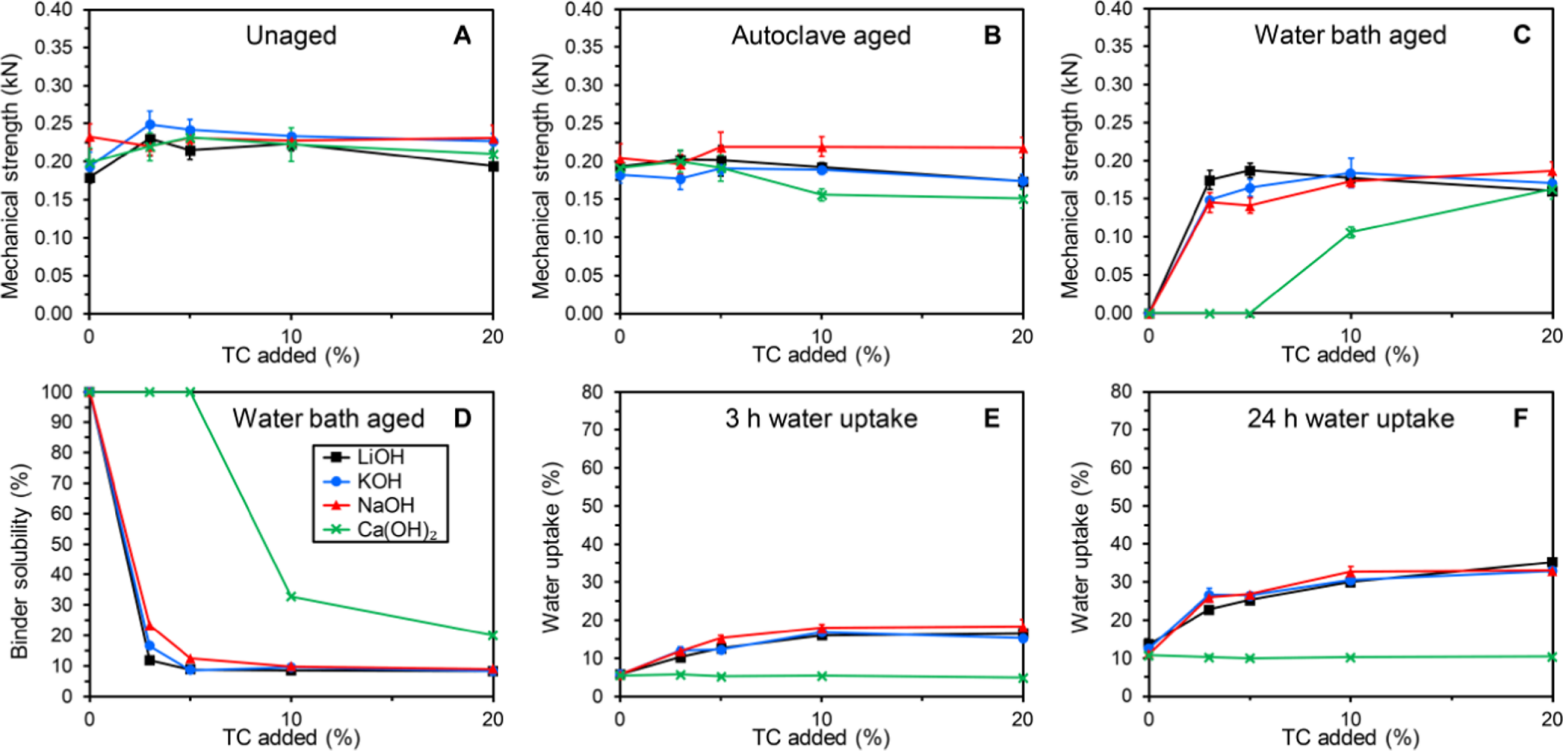
Overview of unaged and aged mechanical strengths (A–C), binder solubility (D), and water uptake properties (E, F) of composite bars made from stone shots and GA120 modified with TC in the presence of LiOH (■, black), KOH (●, blue), NaOH (▲, red), and Ca(OH)_2_ (×, green) at pH 9. Data for mechanical strengths and water uptake are expressed as mean ± standard error (*n* = 5 and *n* = 3, respectively).

The effect of varying the metal cation of the hydroxide base was investigated using GA120 modified with TC as a model system. An overview of the results obtained for bars produced with GA120 modified with 0–20% TC in the presence of NaOH, KOH, LiOH, and Ca(OH)_2_ at pH 9 is shown in Figure 4. The mechanical strengths and binder solubilities previously disclosed^10^ for use of NaOH at pH 9 and previously partly disclosed^9^ for use of Ca(OH)_2_ at pH 9 are included for comparison.

The use of either of the monovalent potassium, sodium, or lithium as counterions generally resulted in comparable binder properties (Figure 4A–F). There were, however, some local differences such as lower water solubility for the use of LiOH at low tannin addition (≤5% tannin addition, Figure 4D). Nonetheless, the fact that these alkali metal hydroxides may be used more or less interchangeably is an advantage to the binder technology. The effect of using the divalent calcium ion as counterions was, on the other hand, very pronounced, most notably on the water bath-aged strengths, water solubility, and water uptakes (Figure 4C–F).^9^ Thus, the water uptake remained virtually constant at the same low level regardless of tannin addition levels when Ca(OH)_2_ was used as the base (Figure 4E,F). For binder compositions including tannin, the water uptake was therefore in the vicinity of only one-third of those obtained when alkali metal hydroxides were used. However, binder compositions with less than 10% tannin disintegrated during the water bath aging treatment (Figure 4C,D). The addition of at least 20% tannin was required to attain water bath-aged strengths comparable to those obtained with alkali metal hydroxides (Figure 4C). The water solubility still remained significantly higher though (Figure 4D). These observations indicate that the presence of divalent cations such as calcium to some degree hampers the formation of strong interactions between gelatin and the tannin components. This may be caused by formation of complexes with calcium ions, whereby fewer reactive sites will be available for interaction between these two components. Indeed, the presence of at least two adjacent hydroxy groups on the phenyl rings in the plant polyphenols is known to enable metal chelation with, for example, calcium.^16^ As an alternative to using high tannin addition levels, it may be envisioned that combinations of, for example, Ca(OH)_2_ and NaOH, or indeed other combinations of di- or polyvalent ions and monovalent ions can be employed to attain suitable balances between low water uptake, low water solubilities, and high water bath-aged strengths.^9^

### Variations in pH

**Figure 5:**
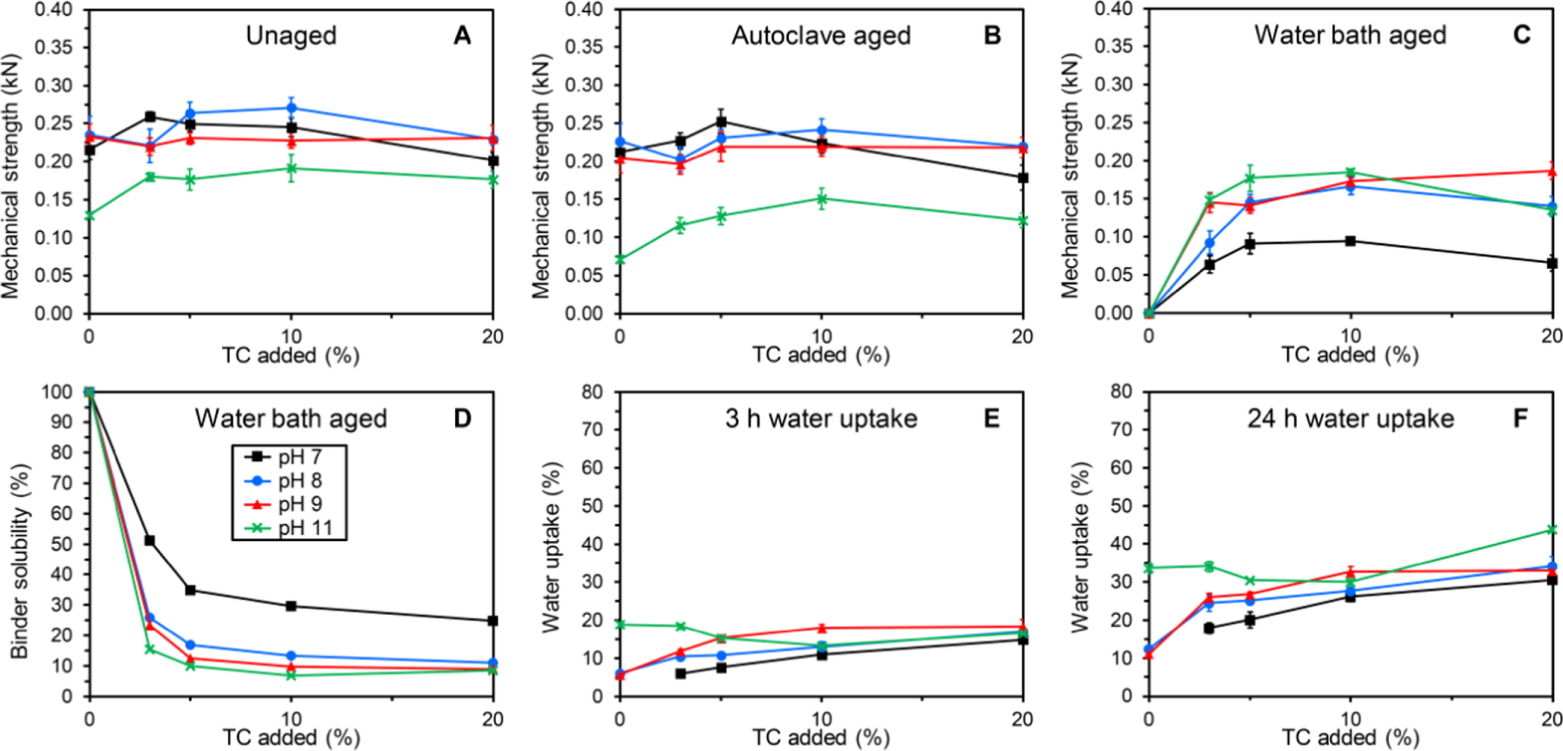
Overview of unaged and aged mechanical strengths (A–C), binder solubility (D), and water uptake properties (E, F) of composite bars made from stone shots and GA120 modified with TC in the presence of NaOH at pH 7 (■, black), pH 8 (●, blue), pH 9 (▲, red), and pH 11 (×, green). Data for the mechanical strengths and water uptake are expressed as mean ± standard error (*n* = 5 and *n* = 3, respectively).

GA120 modified with TC in the presence of NaOH was used as a model system to study the effect of varying the pH of the binder mixtures. An overview of the results obtained for bars produced with GA120 modified with 0–20% TC in the presence of NaOH at pH 7, 8, 9, and 11 is shown in Figure 5. The mechanical strengths and binder solubilities for modification with NaOH at pH 9 were reported previously^10^ and were included for comparison.

As discussed above, one of the two prevalent modification pathways of gelatins with tannins is the formation of covalent bonds under oxidative conditions, which is promoted by basic conditions. To this end, the binder compositions mixed to pH 7 generally displayed significantly lower water bath-aged strengths as well as higher water solubilities than the binder compositions at higher pH (Figure 5C,D). By contrast, the lowest binder solubilities were obtained for the binder compositions mixed at pH 11 (Figure 5D). However, the unaged and, especially, the autoclave-aged strengths were generally significantly lower for these high pH binder compositions than for their counterparts at lower pH (Figure 5A,B). These results indicate that the optimal conditions for modification of the gelatins are situated in the pH range 8–9 (or more broadly from >7 to <11). The general trend observed for the water uptake properties (Figure 5E,F) was an increase in water uptake with increasing pH of the binder mixture (although a few exceptions were observed).

### Further Mechanistic Studies

To gain further insights into the gelatin modification mechanisms occurring under the conditions used herein, where the gelatin–tannin binder mixture is in contact with a stone surface, a set of composite bars were produced under argon in a purpose-built glovebox (see the SI for details). The bars were made using GA120 modified with 10% TC in the presence of NaOH at pH 9 as a representative binder system. For comparison, an analogous set of composite bars were produced under normal air, likewise in the glovebox. The bars produced under argon displayed unaged mechanical strengths almost comparable to the analogous bars made under air (16% weaker). Intriguingly, though, the mechanical strengths of the bars made under argon were 34% lower after the water bath aging process than the corresponding bars made under air, and the water solubility of the binder system was five times higher. The combination of these observations and the results from the above pH studies indicate that the modification pathway based on the formation of covalent bonds under oxidative conditions is important for optimal modification of the gelatin with tannins under the present conditions. However, considerable water bath aging stability remained even when removing oxygen from the reaction environment, which suggests that the complex combination of noncovalent interactions between gelatins and tannins likewise plays a vital role in the modification mechanism.

## Conclusions

The systematic in-depth studies presented herein for the first time of binder systems based on gelatins modified with tannins revealed large and intriguing impacts from varying the nature of the gelatin component (type, gel strength, viscosity, conductivity), the tannin component (hydrolyzable, condensed, source), the metal cation of the hydroxide base (alkali, calcium), and the final pH (7–11). These studies have unveiled a greener binder technology that offers a wide range of possibilities for tailor-making properties toward various application areas. Thus, the combination of high unaged and aged mechanical strengths and low water solubilities is generally favored by the use of gelatins with higher gel strength (and hence, generally higher molecular weights), low-to-mid range tannin additions levels (3–20%), alkali metal hydroxides for pH adjustment, and final pH in the range 8–9 (or more broadly from above 7 to below 11). If desired, comparatively low water uptake properties are favored using higher gel strength type A gelatins, lower tannin addition levels, alkali metal hydroxides for pH adjustment, and lower final pH. Even lower water uptake properties may be obtained using Ca(OH)_2_ in place of alkali metal hydroxides, though at the cost of higher water solubility. High water uptake is, however, desirable in a number of application areas such as precision growing or water management applications, and this is favored using type B gelatins in place of type A gelatins (and lower gel strength gelatins in general), higher tannin addition levels (or indeed, also lower when combined with type B gelatins), and higher final pH. Other gelatin properties such as viscosity and conductivity as well as mixtures of gelatins could also be used to modulate the overall properties of the binder system. High mechanical strength and efficient modification leading to low water solubility of the resulting binder system could be obtained by the use of both hydrolyzable and condensed type tannins. This flexibility toward the tannin type and source is advantageous for binder technology, but complementary studies of well-defined model tannins would be of high interest to establish reliable structure–activity relationships. Intriguingly, significantly lower mechanical strengths after water bath aging and higher water solubilities were observed for bars produced at lower pH ranges or under oxygen-deprived conditions. This indicates that gelatin modification pathways based both on the formation of covalent bonds under oxidative conditions as well as on the complex combination of noncovalent interactions are important for optimal modification of gelatin with the tannin component under the present conditions.

Overall, the detailed studies presented herein represent highly important advances for the understanding and development of greener binder systems based on proteins such as gelatins modified with phenolics such as tannins. This new and highly promising generation of nature-inspired binder technologies is expected to enable the production of greener stone wool and related materials by omitting the use of components that are fossil based and/or toxic in their uncured form and/or require curing temperatures well beyond 200 °C. The latter requirement alone results in adverse environmental effects such as high energy consumption and possibly also emissions that may require post-treatment. On the contrary, the gelatin–tannin-based binder systems studied herein are capable of curing even as low as ambient temperatures and only comprise natural and nontoxic binder components. The capability of curing at ambient temperatures may furthermore serve to reduce or even eliminate production waste streams arising from uncured spots and/or areas in the stone wool. It may also enable the direct manufacture of tailor-made products, possibly even on the installation site. This would reduce another waste stream from cutting and fitting products and improve the performance. The development and application of these highly promising binder systems in the production of greener stone wool products is still in its early stages, but the anticipated reduction in various adverse environmental effects was indeed observed during the production of the prototype stone wool product shown in the Abstract Graphic.

## Experimental Section

### General Experimental Methods

Gelatins GA78 (IMAGEL RL, type A, 78 bloom), GA120 (IMAGEL LA, type A, 120 bloom), GA180 (IMAGEL RA, type A, 180 bloom), GA305 (IMAGEL AP, type A, 305 bloom), GA291v (IMAGEL HP, type A, 291 bloom), GB122 (IMAGEL LB, type B, 122 bloom), GB267 (IMAGEL DP, type B, 267 bloom), and GB278vs (IMAGEL SI, type B, 278 bloom) were obtained from GELITA AG and have a defined cross-linking behavior (see the SI for further details on gelatins). Tannins TC (tannin from chestnut trees, Vinoferm Tannorouge, hydrolysable tannin) and TO (tannin from oak trees, Tannivin Superb Erbslöh, hydrolyzable tannin) were obtained from Brouwland Bvba. TA (tannic acid, gallotannin) was obtained from Sigma-Aldrich. TQ (tannin from quebracho trees, Tannivin Structure, high proanthocyanidin) was obtained from G. Wein GmbH + Co. TG (tannin from grape, Tanin VR Grape, proanthocyanidic tannins) was obtained from Laffort. NaOH, KOH, and LiOH monohydrate were obtained from Sigma-Aldrich. Ca(OH)_2_ was obtained from TCI Europe. For simplicity, these reagents were assumed completely pure. Stone shots formed during the cascade spinning process of a stone melt in the production of stone wool fibers were obtained from a ROCKWOOL production in Denmark and were cleaned and sifted to diameter sizes of 0.25–0.50 mm as previously described.^10^ FUNKTION heat-resistant silicone forms for the manufacture of bars (4 × 5 slots per form; slot top dimension: length = 5.6 cm, width = 2.5 cm; slot bottom dimension: length = 5.3 cm, width = 2.2 cm; slot height = 1.1 cm) were obtained from F&H of Scandinavia A/S. See the SI for further details on general experimental methods.

### Representative Mixing Example for Preparation of Tannin Mixture (TO Mixture)

TO (9.00 g) was added to 0.9 M NaOH (31.5 g) stirred at room temperature. After stirring for at least 30 min further at room temperature, the resulting brownish mixture (pH 9.2) was used in the subsequent experiments. See the SI for details on all tannin mixtures.

### Representative Mixing Example for Gelatin Modified with Tannin (GA120 Modified with 5% TO in the Presence of NaOH at pH 9)

A mixture of GA120 (12.00 g) in water (65.42 g) was stirred at 50 °C for approximately 15–30 min until a clear solution was obtained (pH 4.9). 1M NaOH (5.90 g) was then added (pH 9.1) followed by a portion of the above TO mixture (2.70 g; thus efficiently 0.60 g TO). After stirring for 1–2 min further at 50 °C, the resulting brown mixture (pH 9.0) was used in the subsequent experiments. See the SI for details on all gelatin–tannin mixtures.

### Composite Bars

A 15 wt % binder solution was obtained as described in the representative example above. A sample of the binder solution (16.0 g for GA78/GA120/GA180/GB122; 8.40 g for GA305/GA291v/GB267/GB278vs) was mixed well with shots (80.0 g for GA78/GA120/GA180/GB122; 42.0 g for GA305/GA291v/GB267/GB278vs; preheated to 40 °C and 50 °C when used in combination with GA180 and GA305/GA291v/GB267/GB278vs, respectively). The resulting mixture was then transferred into four slots (GA78/GA120/GA180/GB122) or two slots (GA305/GA291v/GB267/GB278vs) in a silicone form for making bars. Each composite bar was pressed and evened out as required with a plastic spatula to fill out the slot and generate an even bar surface. Around 30 bars were generally produced from each binder composition. The production of a surplus of bars allowed for discarding bars during the various treatment processes due to the presence of visual irregularities such as uneven surfaces, cracks, and/or air pockets created during the manufacturing process. The resulting composite bars were stored at room temperature first for 1–2 days in the form and then for 1–2 days upside down out of the form to cure and dry completely. See the SI for details on further treatments of the bars.

## Supporting Information Available

Further details and studies to Results and Discussion (gelatin viscosity parameter, mixtures of gelatins, contact angle studies); further details to Experimental Section (details on gelatins, further general experimental methods, manufacture of bars under argon, treatment methods of composite bars, and measurement of composite bar properties); and further details on mixing of binder compositions and results for composite bars (PDF)

## Abbreviations Used

GA78: IMAGEL RL (type A gelatin, 78 bloom);
GA120: IMAGEL LA (type A gelatin, 120 bloom);
GA180: IMAGEL RA (type A gelatin, 180 bloom);
GA305: IMAGEL AP (type A gelatin, 305 bloom);
GA291v: IMAGEL HP (type A gelatin, 291 bloom, high viscosity);
GB122: IMAGEL LB (type B gelatin, 122 bloom);
GB267: IMAGEL DP (type B gelatin, 267 bloom);
GB278vs: IMAGEL SI (type B gelatin, 278 bloom, high viscosity and low salt);
TC: tannin from chestnut trees;
TO: tannin from oak trees;
TA: tannic acid;
TQ: tannin from quebracho trees;
TG: tannin from grapes.
