# Supplementary material for: Gelatin–Tannin-Based Greener Binder Technology for Stone Shot and Stone Wool Materials: A Detailed Study
Source: ACS Omega. 2021 Nov 30;6(49):33874–82. doi: 10.1021/acsomega.1c05153 (PMC8674984; doi:10.1021/acsomega.1c05153)
Supplement: Supplementary file 1 — ao1c05153_si_001.pdf [file ao1c05153_si_001.pdf]

Gelatin-Tannin Based Greener Binder Technology for Stone Shot and Stone Wool Materials: A Detailed Study

Thomas Hjelmgaard,[a] Josefine Øgaard Svendsen,[a] Berthold Köhler,[b] Paul Pawelzyk,[b] Dorthe Lybye,[a] Carina Michella Schmücker,[a] Peter Reiter,[b] Matthias Reihmann,[b] Peter Anker Thorsen[a]*

[a] ROCKWOOL International A/S, Hovedgaden 584, Entrance C, 2640 Hedehusene, Denmark

[b] GELITA AG, Uferstrasse 7, 69502 Eberbach, Germany.

* E-mail: thomas.hjelmgaard@rockwool.com

CONTENTS

Further details to Results and Discussion	S2
Variations in gelatins – viscosity parameter	S2
Variations in gelatins – mixtures of gelatins	S3
Variations in gelatins – contact angle studies	S5
Further details to Experimental Section	S7
General experimental methods – details on gelatins	S7
General experimental methods – further details	S7
Manufacture of composite bars under argon	S8
Ageing treatment of composite bars	S9
Measurement of mechanical strengths of composite bars	S9
Measurement of binder content in composite bars	S10
Measurement of water uptake in composite bars	S10
Films for contact angle measurements	S11
Further details on mixing of binder compositions	S12
Further detailed results for composite bars	S28
References	S48

FURTHER DETAILS TO RESULTS AND DISCUSSION

Variations in gelatins – viscosity parameter

In order to investigate the impact of the viscosity parameter of the gelatin component, a comparative study was carried out herein using the type A gelatin GA291v. This gelatin is characterized by a gel strength comparable to that of the type A gelatin GA305 (291 vs. 305 bloom) while the viscosity is significantly higher (5.5 vs. 3.9 mPa×s for a 6.67% aq. solution at 60 °C). An overview of the results obtained for bars produced with GA291v, GA305 and GA120 modified with 0-50% TC in the presence of NaOH at pH 9 is shown in Figure S1. The mechanical strengths and binder solubilities for the use of GA305 and GA120 were reported previously,(S1) and are included for comparison.

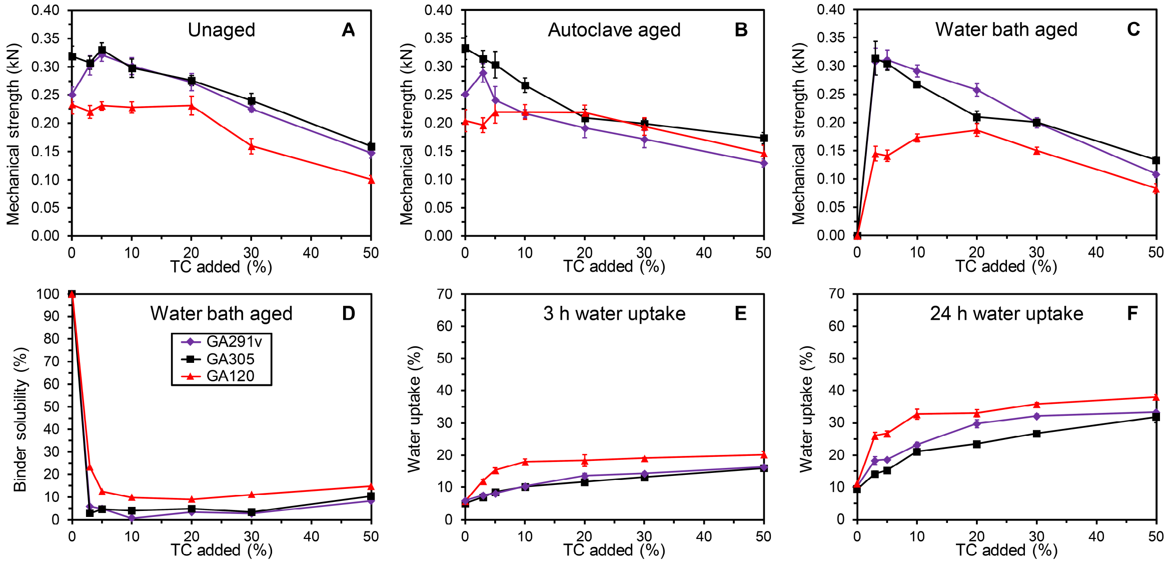

FIGURE S1. Overview of unaged and aged mechanical strength (A-C), binder solubility (D) and water uptake properties (E-F) of composite bars made from stone shots and GA291v (♦, violet), GA305 (■, black), and GA120 (▲, red) modified with TC in the presence of NaOH at pH 9. Data for the mechanical strengths and water uptakes are expressed as mean ± standard error (n = 5 and n = 3, respectively).

When comparing the results obtained for GA291v and GA305, the viscosity generally only had a minor impact on the unaged mechanical strengths of the bars (Fig. S1A). After subjection to autoclave ageing, however, the mechanical strengths of the bars produced with the higher viscosity GA291v were generally somewhat lower than for the analogous bars made with GA305 (Fig. S1B). Interestingly, though, the opposite pattern was obtained after water bath ageing where the bars produced with GA291v generally performed at least as well as the bars produced with GA305 (Fig. S1C). This was also reflected in a slightly lower general solubility of the binder series based on GA291v when compared to GA305 (Fig. S1D). After 3 h, only small differences in water uptake properties were observed between bars made with GA291v and GA305 (Fig. S1E). However, after 24 h, the water uptakes of bars made with GA291v were generally higher than the water uptakes of bars made with GA305 (Fig. S1F). Overall, these results show that the viscosity parameter has a significant impact on the resulting binder properties and this parameter should therefore be considered when developing these gelatin-based binder systems.

Variations in gelatins – mixtures of gelatins

An important effect of increasing the strength and viscosity of the gelatins is that the setting time of the resulting binder system decreases accordingly. While a short setting time may be useful in some applications, a longer setting time may be desirable in others. To this end, adding a small amount of high strength gelatins to lower strength gelatins may result in significant strength effects while the setting time remains unchanged. The effect of substituting 10% of GA120 with GA305 (high strength type A gelatin) or GB278vs (high strength type B gelatin) was therefore investigated. An overview of the results obtained for bars produced with GA120/GA305 90:10, GA120/GB278vs 90:10, GA305 and GA120 modified with 0-20% TC

in the presence of NaOH at pH 9 is shown in Figure S2. The mechanical strengths and binder solubilities for use of for GA305 and GA120 were reported previously,(S1) and are included for comparison.

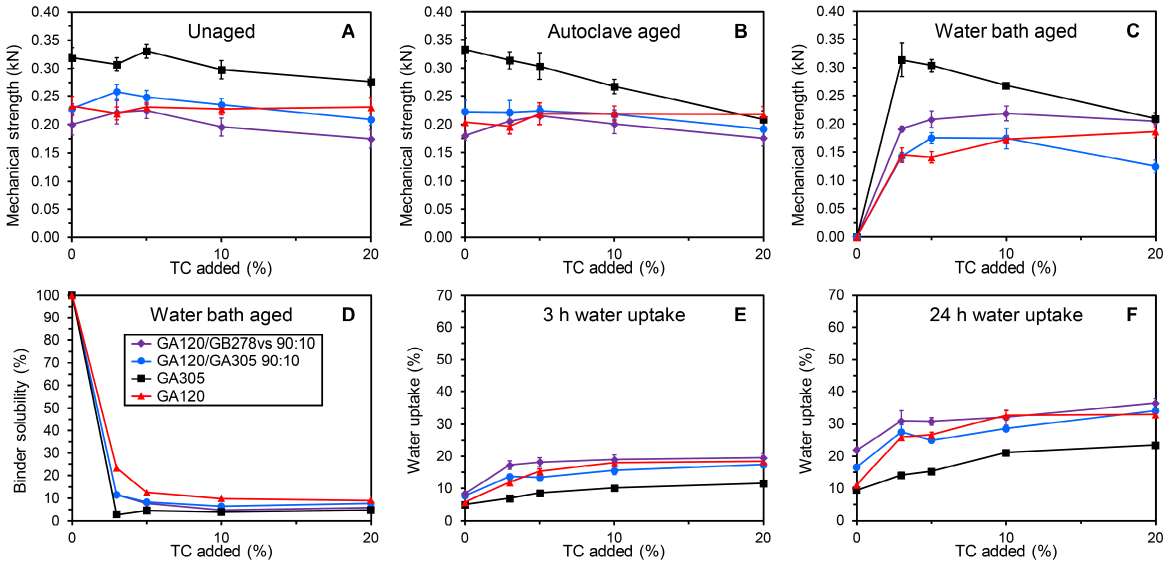

FIGURE S2. Overview of unaged and aged mechanical strength (A-C), binder solubility (D) and water uptake properties (E-F) of composite bars made from stone shots and GA120/GB278vs 90:10 (♦, violet), GA120/GA305 90:10 (●, blue), GA305 (■, black), and GA120 (▲, red) modified with TC in the presence of NaOH at pH 9. Data for the mechanical strengths and water uptakes are expressed as mean ± standard error (n = 5 and n = 3, respectively).

As expected, the substitution of 10% of GA120 with the higher strength gelatins GA305 or GA278vs resulted in no apparent changes to the binder setting time during manufacture of the composite bars compared to the use of GA120 as the sole gelatin component. The most significant strength increase compared to use of GA120 only was observed for substitution with 10% GB278vs after water bath ageing (Fig. S2C). While the inclusion of 10% GB278vs thus provided better strength results after water bath ageing than inclusion of 10% GA305 (Fig. S2C), substitution with 10% GA305 appeared to yield the best results of the two in terms of

unaged and autoclave aged strengths, providing some strength improvements at low tannin content range (2.5-5%, Fig. S2A-B). Interestingly, the inclusion of the two high strength gelatins had a significant impact on the binder solubility which generally decreased to levels closer to those obtained for the use of GA305 or GB278vs only (Fig. S2D). As could be expected, the water uptakes generally increased slightly as a result of substitution with 10% of the type B gelatin GB278vs, especially at low tannin contents (Fig. S2E-F). Only minor effects were observed as a result of substitution with 10% of the type A gelatin GA305 (Fig. S2E-F). Overall, these results demonstrated that significant effects on the properties of the gelatin-based binder systems may be obtained by mixing gelatins with different characteristics such as gel strength and type.

Variations in gelatins – contact angle studies

In order to study the wettability of the gelatine-based binder systems both in the presence and in the absence of a stone surface, films were produced from GA120 and GB122 modified with 0%, 10%, and 50% TC in the presence of NaOH at pH 9. The resulting contact angles measured 10 seconds after application of a droplet of water are listed in Table S1 and illustrated in Figure S3.

Entry	1	2	3	4	5	6
**Binder composition**						
GA120	100	100	100	-	-	-
GB122	-	-	-	100	100	100
TC (%-wt. of gelatin)	0	10	50	0	10	50
**Film results**						
Avg. contact angle (°, n = 3)	54.4	47.5	46.5	52.7	46.1	45.6
Standard error (%)	2.2	1.1	3.7	2.5	1.1	0.9

TABLE S1. Results for contact angle measurements.

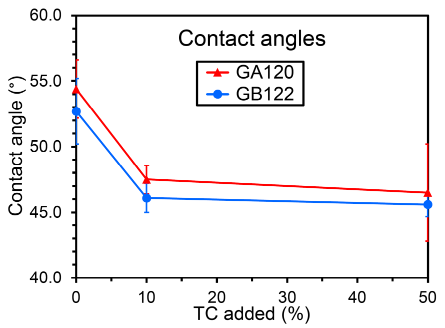

FIGURE S3. Contact angles measured for films made from GA120 (▲, red) and GB122 (●, blue), modified with TC and NaOH at pH 9.

The measurements on films confirmed the results obtained from the water uptake measurements on composite bars produced with the analogous binder systems. Thus, the gelatine-based binder systems generally became more hydrophilic with increasing amounts of TC added and the most significant increase in hydrophilicity was observed in the 0-10% TC range. Furthermore, the binder compositions comprising the type B gelatin GB122 were more hydrophilic than the corresponding binder compositions based on the type A gelatin GA120.

FURTHER DETAILS TO EXPERIMENTAL SECTION

General experimental methods – details on gelatins

Table S2 summarizes the properties of the gelatins used herein.

**Gelatin**	**Commercial name**	**Type**	**Bloom**	**Viscosity** (mPa×s at 6.67%/60°C)	**Loss on drying** (%)	**pH** (at 6.67%/ 60 °C)	**Conductivity** (µS/cm at 1.00%/30 °C)
GA78	IMAGEL RL	A (porcine)	78	1.67	11.8	4.92	-
GA120	IMAGEL LA	A (porcine)	120	2.06	11.6	5.08	-
GA180	IMAGEL RA	A (porcine)	180	3.00	11.9	5.13	-
GA305	IMAGEL AP	A (porcine)	305	3.90	10.8	5.31	155
GA291v	IMAGEL HP	A (porcine)	291	5.53	10.3	5.58	156
GB122	IMAGEL LB	B (bovine)	122	2.31	11.9	5.32	61
GB267	IMAGEL DP	B (bovine)	267	2.89	9.6	5.46	63
GB278vs	IMAGEL SI	B (bovine)	278	4.41	10.6	5.08	10

TABLE S2. Properties of gelatins.

General experimental methods – further details

Measurements of pH were performed using a Mettler Toledo SevenCompactTM S220 pH meter equipped with a Mettler Toledo InLab Expert Pro-ISM pH electrode and temperature probe. Contact angle measurements were conducted with a DataPhysics OCA 15+ with en electronic syringe module and a film support rig. Three-point bending tests of composite bars were recorded on a Bent Tram SUT 3000/520 test machine (test speed: 10.0 mm/min; rupture level: 50 N; nominal strength: 30 N/mm^2; support distance: 40 mm; max deflection 20 mm; nominal E-modulus 10000 N/mm^2). The bars were placed with the "top face" up (*i.e.* the face with the dimensions length = 5.6 cm, width = 2.5 cm) in the machine. New tin foil containers

for use in measurement of binder contents of composite bars were heat-treated at 590 °C for 15 minutes prior to use to remove all organics.

Manufacture of composite bars under argon

The required reagents (GA120, TC and stone shots) were weighed off in the appropriately sized containers with magnetic stirring bars when relevant. These containers as well as further required equipment (magnetic stirrer, empty water bath, weight, bar form, spatulas and pipettes) were then placed in a purpose-built glove box (60×50×40 cm width/depth/height). Argon was bubbled through appropriately sized containers containing the required aqueous solutions (1M NaOH as well as deionized water) for 30-45 mins. The containers were then closed tight and were also placed in the glove box. The glove box was then sealed tight except for a small 4 mm diameter exit hole in the top lid of the box. From then on, the glove box was kept under a constant argon stream. The argon stream was set to a high rate during the first few hours as well as when working in the box and for an hour after working in the box. At all other times the argon stream was set to a slow rate (so that a weak argon stream exiting the top lid hole could continuously be detected). Argon was passed through the glove box for a day during which time the different solid components (GA120, TC and stone shots) were stirred gently with plastic spatulas a few times. A sensitive CO_2 measurement equipment placed inside the box indicated CO_2 levels below the measurement limits. Water was then added to the water bath and the heating was turned on. Once the water bath had reached 50 °C, the reagent solutions were prepared. After stirring for 30 min, the finalized binder solution was prepared and 20 composite bars were produced as described in the article. The bars were carefully taken out of the containers after 1 day, turned upside down, and were then left for 2 days further to

cure and dry completely. The bars were taken out of the box and a selection of the bars were subjected to water bath ageing after 2 h.

To enable direct comparison with composite bars made in the presence of oxygen, a set of 20 bars were manufactured using a similar procedure, this time applying an air stream rather than an argon stream to the glove box. For convenience, the reagent solutions and finalized binder solutions were prepared outside the glove box. The binder solution was also added to the stone shots outside the glove box, but the rest of the procedure was then carried out inside the glove box as described above.

Ageing treatment of composite bars

Ageing treatment of composite bars was performed by subjecting the bars to autoclave treatment (15 min / 120 °C / 1.2 bar) or water bath treatment (3 h / 80 °C) followed by cooling to room temperature and drying for 2-3 days.

Measurement of mechanical strengths of composite bars

The maximum load force required to break composite bars was recorded in a three-point bending test. For each data point, an average value was calculated on the basis of five bars that had been subjected to identical treatment.

Measurement of binder content in composite bars

The binder content in the composite bars was measured in small tin foil containers by treatment at 590 °C. The tin foil container was weighed and four bars (usually after being broken in the three-point bending test) were placed into the tin foil container. The ensemble was weighed and was then heat-treated at 590 °C for 30 minutes. After cooling to room temperature, the weight was recorded again and the binder content was calculated as percentage of the stone shots.

Measurement of water uptake in composite bars

The water uptake of the binders was measured by weighing three bars and then submerging the bars in water (approx. 250 mL) in a beaker (565 mL, bottom Ø = 9.5 cm; top Ø = 10.5 cm; height = 7.5 cm) for 3 h or 24 h. The bars were placed next to each other on the bottom of the beaker with the "top face" down (*i.e.* the face with the dimensions length = 5.6 cm, width = 2.5 cm). After the designated amount of time, the bars were lifted up one by one and allowed to drip off for one minute. The bars were held (gently) with the length side almost vertical so that the droplets would drip from a corner of the bar. The bars were then weighed and the water absorption was calculated as the percentage in weight increase of the start weight. For each data point, an average value was calculated on the basis of three bars that had been subjected to identical treatment.

Films for contact angle measurements

15%-wt. binder solutions were obtained as described in the representative example in the article. The binder solutions were coated on top of polyethylene terephthalate films using an Erichsen COATMASTER 509 MC equipped with a film applicator set at 500 µm. The films were dried and conditioned for at least two weeks in a climatized room at 22 °C and 50% rh. The smooth bottom-side of the films was used for contact angle measurements. Measurement of contact angles was performed 10 sec after application of the water droplet in order to avoid any potential influence from swelling.

FURTHER DETAILS ON MIXING OF BINDER COMPOSITIONS

Mixing of binder compositions comprising GA78, GA120, GA180, GA305 and GA291v modified with TC in the presence of NaOH at pH 9

Mixing details about binder compositions comprising GA78, GA120, GA180 and GA305 modified with TC in the presence of NaOH at pH 9 have been published previously.[(S1)] The mixing details of binder compositions comprising GA291v modified with TC in the presence of NaOH at pH 9 are listed in Table S3. The binder compositions were mixed using procedures analogous to the representative examples described in the article, using 1.0 M NaOH for mixing of the TC mixture (deep red-brown).

**Entry**	**1**	**2**	**3**	**4**	**5**	**6**	**7**
**Binder composition**							
GA291v	100	100	100	100	100	100	100
TC (%-wt. of gelatin)	0	3	5	10	20	30	50
**Mixing of binder composition**							
GA291v (g)	12.00	12.00	12.00	12.00	12.00	11.00	9.50
Water (g)	65.92	66.95	67.47	69.63	73.11	70.48	66.50
1M NaOH (g)	2.95	2.95	2.95	2.95	2.95	2.70	2.30
pH	9.1	9.1	9.2	9.2	9.1	9.3	9.2
TC mixture (g)	0	1.62	2.70	5.40	10.80	14.85	21.38
pH	-	9.1	9.2	9.2	9.2	9.4	9.3
**Calculated binder composition**							
Binder concentration (%-wt. in water)	15.0	15.0	15.0	15.0	15.0	15.0	15.0
GA291v (%-wt. of gelatin/ %-wt. of all non-water components)	100.0/ 99.1	100.0/ 95.8	100.0/ 93.8	100.0/ 89.1	100.0/ 80.9	100.0/ 74.1	100.0/ 63.4
TC (%-wt. of gelatin/ %-wt. of all non-water components)	0/ 0	3.0/ 2.9	5.0/ 4.7	10.0/ 8.9	20.0/ 16.2	30.0/ 22.2	50.0/ 31.7
NaOH (%-wt. of gelatin/ %-wt. of all non-water components)	0.9/ 0.9	1.4/ 1.3	1.6/ 1.5	2.3/ 2.0	3.6/ 2.9	5.0/ 3.7	7.7/ 4.9

TABLE S3.

Mixing of binder compositions comprising GB122 modified with TC in the presence of NaOH at pH 9

The mixing details of binder compositions comprising GB122 modified with TC in the presence of NaOH at pH 9 are listed in Table S4. The binder compositions were mixed using procedures analogous to the representative examples described in the article, using 1.0 M NaOH for mixing of the TC mixture (deep red-brown).

**Entry**	**1**	**2**	**3**	**4**	**5**	**6**	**7**
**Binder composition**							
GB122	100	100	100	100	100	100	100
TC (%-wt. of gelatin)	0	3	5	10	20	30	50
**Mixing of binder composition**							
GB122 (g)	12.00	12.00	12.00	12.00	12.00	11.00	9.50
Water (g)	64.92	66.43	66.95	68.54	72.51	69.88	65.90
1M NaOH (g)	3.85	3.85	3.85	3.85	3.85	3.73	3.14
pH	9.2	9.1	9.0	9.0	9.0	8.8	9.0
TC mixture (g)	0	1.62	2.70	5.40	10.80	14.85	21.38
pH	-	9.0	9.0	9.0	9.0	9.0	9.1
**Calculated binder composition**							
Binder concentration (%-wt. in water)	15.0	15.0	15.0	15.1	15.0	15.0	15.0
GB122 (%-wt. of gelatin/<br>%-wt. of all non-water components)	100.0/<br>98.8	100.0/<br>95.6	100.0/<br>93.5	100.0/<br>88.8	100.0/<br>80.7	100.0/<br>73.9	100.0/<br>63.3
TC (%-wt. of gelatin/<br>%-wt. of all non-water components)	0/<br>0	3.0/<br>2.9	5.0/<br>4.7	10.0/<br>8.9	20.0/<br>16.1	30.0/<br>22.2	50.0/<br>31.6
NaOH (%-wt. of gelatin/<br>%-wt. of all non-water components)	1.2/<br>1.2	1.6/<br>1.6	1.9/<br>1.8	2.6/<br>2.3	3.9/<br>3.2	5.3/<br>4.0	8.0/<br>5.1

TABLE S4.

Mixing of binder compositions comprising GB267 modified with TC in the presence of NaOH at pH 9

The mixing details of binder compositions comprising GB267 modified with TC in the presence of NaOH at pH 9 are listed in Table S5. The binder compositions were mixed using procedures analogous to the representative examples described in the article, using 1.0 M NaOH for mixing of the TC mixture (deep red-brown).

**Entry**	**1**	**2**	**3**	**4**	**5**	**6**	**7**
**Binder composition**							
GB267	100	100	100	100	100	100	100
TC (%-wt. of gelatin)	0	3	5	10	20	30	50
**Mixing of binder composition**							
GB267 (g)	12.00	12.00	12.00	12.00	12.00	11.00	9.50
Water (g)	65.42	66.95	67.47	69.08	73.11	70.48	66.50
1M NaOH (g)	3.30	3.30	3.30	3.30	3.30	3.00	2.85
pH	9.1	9.1	9.0	9.1	9.0	9.0	9.0
TC mixture (g)	0	1.62	2.70	5.40	10.80	14.85	21.38
pH	-	9.0	9.0	9.1	9.1	9.0	9.1
**Calculated binder composition**							
Binder concentration (%-wt. in water)	15.0	14.9	15.0	15.1	15.0	15.0	15.0
GB267 (%-wt. of gelatin/ %-wt. of all non-water components)	100.0/ 99.0	100.0/ 95.7	100.0/ 93.7	100.0/ 89.0	100.0/ 80.8	100.0/ 74.0	100.0/ 63.3
TC (%-wt. of gelatin/ %-wt. of all non-water components)	0/ 0	3.0/ 2.9	5.0/ 4.7	10.0/ 8.9	20.0/ 16.2	30.0/ 22.2	50.0/ 31.7
NaOH (%-wt. of gelatin/ %-wt. of all non-water components)	1.1/ 1.0	1.5/ 1.4	1.7/ 1.6	2.4/ 2.1	3.8/ 3.0	5.1/ 3.8	7.9/ 5.0

TABLE S5.

Mixing of binder compositions comprising GB278vs modified with TC in the presence of NaOH at pH 9

The mixing details of binder compositions comprising GB278vs modified with TC in the presence of NaOH at pH 9 are listed in Table S6. The binder compositions were mixed using procedures analogous to the representative examples described in the article, using 1.0 M NaOH for mixing of the TC mixture (deep red-brown).

**Entry**	**1**	**2**	**3**	**4**	**5**	**6**	**7**
**Binder composition**							
GB278vs	100	100	100	100	100	100	100
TC (%-wt. of gelatin)	0	3	5	10	20	30	50
**Mixing of binder composition**							
GB278vs (g)	12.00	12.00	12.00	12.00	12.00	11.00	9.50
Water (g)	64.43	65.92	66.43	68.54	71.92	69.29	65.90
1M NaOH (g)	4.55	4.55	4.55	4.55	4.55	4.17	3.60
pH	9.1	9.3	9.2	9.2	9.1	9.3	9.1
TC mixture (g)	0	1.62	2.70	5.40	10.80	14.85	21.38
pH	-	9.2	9.2	9.2	9.1	9.2	9.1
**Calculated binder composition**							
Binder concentration (%-wt. in water)	15.0	15.0	15.0	15.1	15.0	15.0	15.0
GB278vs (%-wt. of gelatin/ %-wt. of all non-water components)	100.0/ 98.6	100.0/ 95.4	100.0/ 93.3	100.0/ 88.6	100.0/ 80.5	100.0/ 73.8	100.0/ 63.2
TC (%-wt. of gelatin/ %-wt. of all non-water components)	0/ 0	3.0/ 2.9	5.0/ 4.7	10.0/ 8.9	20.0/ 16.1	30.0/ 22.1	50.0/ 31.6
NaOH (%-wt. of gelatin/ %-wt. of all non-water components)	1.5/ 1.4	1.9/ 1.8	2.1/ 2.0	2.8/ 2.5	4.2/ 3.3	5.5/ 4.1	8.2/ 5.2

TABLE S6.

Mixing of binder compositions comprising GA120/GA305 90:10 modified with TC in the presence of NaOH at pH 9

The mixing details of binder compositions comprising GA120/GA305 90:10 modified with TC in the presence of NaOH at pH 9 are listed in Table S7. The binder compositions were mixed using procedures analogous to the representative examples described in the article, using 1.0 M NaOH for mixing of the TC mixture (deep red-brown).

**Entry**	**1**	**2**	**3**	**4**	**5**
**Binder composition**					
GA120 + GA305	100	100	100	100	100
GA120 (%-wt. of gelatin)	90	90	90	90	90
GA305 (%-wt. of gelatin)	10	10	10	10	10
TC (%-wt. of gelatin)	0	3	5	10	20
**Mixing of binder composition**					
GA120 (g)	10.80	10.80	10.80	10.80	9.90
GA305 (g)	1.20	1.20	1.20	1.20	1.10
Water (g)	63.95	64.92	65.92	67.47	65.39
1M NaOH (g)	5.30	5.30	5.30	5.30	5.10
pH	9.0	9.1	9.0	9.0	9.1
TC mixture (g)	0	1.62	2.70	5.40	9.90
pH	-	9.0	9.0	8.9	8.9
**Calculated binder composition**					
Binder concentration (%-wt. in water)	15.0	15.0	15.0	15.0	15.0
GA120 + GA305 (%-wt. of gelatin/ %-wt. of all non-water components)	100.0/ 98.3	100.0/ 95.1	100.0/ 93.1	100.0/ 88.5	100.0/ 80.3
GA120 (%-wt. of gelatin/ %-wt. of all non-water components)	90.0/ 88.5	90.0/ 85.6	90.0/ 83.8	90.0/ 79.6	90.0/ 72.3
GA305 (%-wt. of gelatin/ %-wt. of all non-water components)	10.0/ 9.8	10.0/ 9.5	10.0/ 9.3	10.0/ 8.8	10.0/ 8.0
TC (%-wt. of gelatin/ %-wt. of all non-water components)	0/ 0	3.0/ 2.9	5.0/ 4.7	10.0/ 8.8	20.0/ 16.1
NaOH (%-wt. of gelatin/ %-wt. of all non-water components)	1.7/ 1.7	2.1/ 2.0	2.4/ 2.2	3.0/ 2.7	4.5/ 3.6

TABLE S7.

Mixing of binder compositions comprising GA120/GB278vs 90:10 modified with TC in the presence of NaOH at pH 9

The mixing details of binder compositions comprising GA120/GB278vs 90:10 modified with TC in the presence of NaOH at pH 9 are listed in Table S8. The binder compositions were mixed using procedures analogous to the representative examples described in the article, using 1.0 M NaOH for mixing of the TC mixture (deep red-brown).

Entry	1	2	3	4	5
**Binder composition**					
GA120 + GB278vs	100	100	100	100	100
GA120 (%-wt. of gelatin)	90	90	90	90	90
GB278vs (%-wt. of gelatin)	10	10	10	10	10
TC (%-wt. of gelatin)	0	3	5	10	20
**Mixing of binder composition**					
GA120 (g)	10.80	10.80	10.80	10.80	9.90
GB278vs (g)	1.20	1.20	1.20	1.20	1.10
Water (g)	63.95	64.92	65.92	67.47	65.39
1M NaOH (g)	5.65	5.65	5.65	5.65	5.20
pH	9.0	9.0	9.0	9.0	9.0
TC mixture (g)	0	1.62	2.70	5.40	9.90
pH	-	9.0	9.0	8.9	8.9
**Calculated binder composition**					
Binder concentration (%-wt. in water)	15.0	15.0	15.0	15.0	15.0
GA120 + GB278vs (%-wt. of gelatin/ %-wt. of all non-water components)	100.0/ 98.2	100.0/ 95.0	100.0/ 93.0	100.0/ 88.4	100.0/ 80.3
GA120 (%-wt. of gelatin/ %-wt. of all non-water components)	90.0/ 88.4	90.0/ 85.5	90.0/ 83.7	90.0/ 79.5	90.0/ 72.3
GB278vs (%-wt. of gelatin/ %-wt. of all non-water components)	10.0/ 9.8	10.0/ 9.5	10.0/ 9.3	10.0/ 8.8	10.0/ 8.0
TC (%-wt. of gelatin/ %-wt. of all non-water components)	0/ 0	3.0/ 2.9	5.0/ 4.7	10.0/ 8.8	20.0/ 16.1
NaOH (%-wt. of gelatin/ %-wt. of all non-water components)	1.8/ 1.8	2.2/ 2.1	2.5/ 2.3	3.2/ 2.8	4.5/ 3.6

TABLE S8.

Mixing of binder compositions comprising GA120 modified with TA in the presence of NaOH at pH 9

The mixing details of binder compositions comprising GA120 modified with TA in the presence of NaOH at pH 9 are listed in Table S9. The binder compositions were mixed using procedures analogous to the representative examples described in the article, using 1.5 M NaOH for mixing of the TA mixture (green-brownish, pH 9.2).

**Entry**	**1**	**2**	**3**	**4**	**5**	**6**	**7**
**Binder composition**							
GA120	100	100	100	100	100	100	100
TA (%-wt. of gelatin)	0	3	5	10	20	30	50
**Mixing of binder composition**							
GA120 (g)	14.00	12.00	12.00	12.00	12.00	11.00	9.50
Water (g)	75.17	64.43	65.42	66.95	70.76	68.14	64.14
1M NaOH (g)	5.16	6.22	6.43	6.75	7.49	7.70	8.26
pH	9.1	9.4	9.5	9.8	10.0	10.4	11.1
TA mixture (g)	0	1.62	2.70	5.40	10.80	14.85	21.38
pH	-	9.3	9.3	9.4	9.3	9.3	9.3
**Calculated binder composition**							
Binder concentration (%-wt. in water)	15.1	15.0	15.0	15.0	15.0	15.0	15.0
GA120 (%-wt. of gelatin/ %-wt. of all non-water components)	100.0/ 98.6	100.0/ 94.7	100.0/ 95.2	100.0/ 87.6	100.0/ 79.1	100.0/ 72.1	100.0/ 61.3
TA (%-wt. of gelatin/ %-wt. of all non-water components)	0/ 0	3.0/ 2.8	5.0/ 4.6	10.0/ 8.8	20.0/ 15.8	30.0/ 21.6	50.0/ 30.6
NaOH (%-wt. of gelatin/ %-wt. of all non-water components)	1.4/ 1.4	2.6/ 2.5	3.1/ 2.8	4.1/ 3.6	6.4/ 5.0	8.6/ 6.2	13.3/ 8.1

TABLE S9.

Mixing of binder compositions comprising GA120 modified with TG in the presence of NaOH at pH 9

The mixing details of binder compositions comprising GA120 modified with TG in the presence of NaOH at pH 9 are listed in Table S10. The binder compositions were mixed using procedures analogous to the representative examples described in the article, using 0.7 M NaOH for mixing of the TG mixture (brownish, slightly hazy, pH 9.0).

**Entry**	**1**	**2**	**3**	**4**	**5**	**6**	**7**
**Binder composition**							
GA120	100	100	100	100	100	100	100
TG (%-wt. of gelatin)	0	3	5	10	20	30	50
**Mixing of binder composition**							
GA120 (g)	14.00	12.00	12.00	12.00	12.00	11.00	9.50
Water (g)	75.17	64.92	65.92	67.47	70.76	68.14	64.14
1M NaOH (g)	5.16	5.20	5.20	5.20	5.20	4.85	4.10
pH	9.1	9.3	9.2	9.2	9.3	9.2	9.1
TG mixture (g)	0	1.62	2.70	5.40	10.80	14.85	21.38
pH	-	9.1	9.0	9.0	9.0	9.0	9.0
**Calculated binder composition**							
Binder concentration (%-wt. in water)	15.1	15.0	15.0	15.0	15.0	15.0	15.0
GA120 (%-wt. of gelatin/ %-wt. of all non-water components)	100.0/ 98.6	100.0/ 95.3	100.0/ 93.3	100.0/ 88.8	100.0/ 80.9	100.0/ 74.3	100.0/ 63.9
TG (%-wt. of gelatin/ %-wt. of all non-water components)	0/ 0	3.0/ 2.9	5.0/ 4.7	10.0/ 8.9	20.0/ 16.2	30.0/ 22.3	50.0/ 32.0
NaOH (%-wt. of gelatin/ %-wt. of all non-water components)	1.4/ 1.4	2.0/ 1.9	2.1/ 2.0	2.6/ 2.3	3.6/ 2.9	4.6/ 3.4	6.4/ 4.1

TABLE S10.

Mixing of binder compositions comprising GA120 modified with TO in the presence of NaOH at pH 9

The mixing details of binder compositions comprising GA120 modified with TO in the presence of NaOH at pH 9 are listed in Table S11. The binder compositions were mixed using procedures analogous to the representative examples described in the article.

**Entry**	**1**	**2**	**3**	**4**	**5**	**6**	**7**
**Binder composition**							
GA120	100	100	100	100	100	100	100
TO (%-wt. of gelatin)	0	3	5	10	20	30	50
**Mixing of binder composition**							
GA120 (g)	14.00	12.00	12.00	12.00	12.00	11.00	9.50
Water (g)	75.17	64.92	65.42	67.47	70.76	68.14	64.72
1M NaOH (g)	5.16	5.90	5.90	5.90	5.90	5.60	4.65
pH	9.1	9.0	9.1	9.1	9.1	9.1	9.1
TO mixture (g)	0	1.62	2.70	5.40	10.80	14.85	21.38
pH	-	9.0	9.0	9.0	9.0	9.1	9.0
**Calculated binder composition**							
Binder concentration (%-wt. in water)	15.1	15.0	15.0	15.0	15.0	15.0	15.0
GA120 (%-wt. of gelatin/ %-wt. of all non-water components)	100.0/ 98.6	100.0/ 95.0	100.0/ 93.0	100.0/ 88.4	100.0/ 80.4	100.0/ 73.7	100.0/ 63.3
TO (%-wt. of gelatin/ %-wt. of all non-water components)	0/ 0	3.0/ 2.9	5.0/ 4.7	10.0/ 8.8	20.0/ 16.1	30.0/ 22.1	50.0/ 31.7
NaOH (%-wt. of gelatin/ %-wt. of all non-water components)	1.4/ 1.4	2.3/ 2.1	2.5/ 2.3	3.1/ 2.7	4.3/ 3.5	5.6/ 4.1	8.0/ 5.0

TABLE S11.

Mixing of binder compositions comprising GA120 modified with TQ in the presence of NaOH at pH 9

The mixing details of binder compositions comprising GA120 modified with TQ in the presence of NaOH at pH 9 are listed in Table S12. The binder compositions were mixed using procedures analogous to the representative examples described in the article, using 0.5 M NaOH for mixing of the TQ mixture (brownish, pH 9.2).

**Entry**	**1**	**2**	**3**	**4**	**5**	**6**	**7**
**Binder composition**							
GA120	100	100	100	100	100	100	100
TQ (%-wt. of gelatin)	0	3	5	10	20	30	50
**Mixing of binder composition**							
GA120 (g)	14.00	12.00	12.00	12.00	12.00	11.00	9.50
Water (g)	75.17	64.43	64.92	66.43	69.63	67.01	63.02
1M NaOH (g)	5.16	5.90	6.00	6.00	6.00	5.50	4.75
pH	9.1	9.1	9.1	9.1	9.1	9.1	9.1
TQ mixture (g)	0	1.62	2.70	5.40	10.80	14.85	21.38
pH	-	9.1	9.0	9.0	9.1	9.0	9.0
**Calculated binder composition**							
Binder concentration (%-wt. in water)	15.1	15.0	15.0	15.0	15.0	15.0	15.0
GA120 (%-wt. of gelatin/ %-wt. of all non-water components)	100.0/ 98.6	100.0/ 95.1	100.0/ 93.2	100.0/ 88.8	100.0/ 81.1	100.0/ 76.4	100.0/ 64.4
TQ (%-wt. of gelatin/ %-wt. of all non-water components)	0/ 0	3.0/ 2.9	5.0/ 4.7	10.0/ 8.9	20.0/ 16.2	30.0/ 22.4	50.0/ 32.2
NaOH (%-wt. of gelatin/ %-wt. of all non-water components)	1.4/ 1.4	2.1/ 2.0	2.3/ 2.1	2.6/ 2.3	3.3/ 2.7	4.0/ 3.0	5.4/ 3.4

TABLE S12.

Mixing of binder compositions comprising GA120 modified with TC in the presence of KOH at pH 9

The mixing details of binder compositions comprising GA120 modified with TC in the presence of KOH at pH 9 are listed in Table S13. The binder compositions were mixed using procedures analogous to the representative examples described in the article, using 1.0 M KOH both for pH adjustment and for mixing of the TC mixture (deep red-brown, traces of precipitates).

**Entry**	**1**	**2**	**3**	**4**	**5**
**Binder composition**					
GA120	100	100	100	100	100
TC (%-wt. of gelatin)	0	3	5	10	20
**Mixing of binder composition**					
GA120 (g)	12.00	12.00	12.00	12.00	11.00
Water (g)	64.92	66.43	66.95	69.08	67.01
1M KOH (g)	4.70	4.70	4.70	4.70	4.30
pH	9.2	9.2	9.1	9.1	9.1
TC mixture (g)	0	1.62	2.70	5.40	9.90
pH	-	9.1	9.1	9.0	8.9
**Calculated binder composition**					
Binder concentration (%-wt. in water)	15.0	15.0	15.0	15.0	15.0
GA120 (%-wt. of gelatin/	100.0/	100.0/	100.0/	100.0/	100.0/
%-wt. of all non-water components)	98.0	94.7	92.6	87.8	79.5
TC (%-wt. of gelatin/	0/	3.0/	5.0/	10.0/	20.0/
%-wt. of all non-water components)	0	2.8	4.6	8.8	15.9
KOH (%-wt. of gelatin/	2.1/	2.6/	3.0/	3.9/	5.8/
%-wt. of all non-water components)	2.0	2.5	2.8	3.5	4.6

TABLE S13.

Mixing of binder compositions comprising GA120 modified with TC in the presence of LiOH at pH 9

The mixing details of binder compositions comprising GA120 modified with TC in the presence of LiOH at pH 9 are listed in the Table S14. The binder compositions were mixed using procedures analogous to the representative examples described in the article, using 1.0 M LiOH both for pH adjustment and for mixing of the TC mixture (deep red-brown, pH 9.2).

**Entry**	**1**	**2**	**3**	**4**	**5**
**Binder composition**					
GA120	100	100	100	100	100
TC (%-wt. of gelatin)	0	3	5	10	20
**Mixing of binder composition**					
GA120 (g)	12.00	12.00	12.00	12.00	11.00
Water (g)	64.43	65.42	65.92	67.47	64.86
1M LiOH (g)	4.30	4.30	4.30	4.30	3.95
pH	9.2	9.1	9.1	9.1	9.2
TC mixture (g)	0	1.62	2.70	5.40	9.90
pH	-	9.1	9.2	9.1	9.2
**Calculated binder composition**					
Binder concentration (%-wt. in water)	15.0	15.0	15.0	15.0	15.0
GA120 (%-wt. of gelatin/ %-wt. of all non-water components)	100.0/ 99.2	100.0/ 96.1	100.0/ 94.1	100.0/ 89.6	100.0/ 81.6
TC (%-wt. of gelatin/ %-wt. of all non-water components)	0/ 0	3.0/ 2.9	5.0/ 4.7	10.0/ 9.0	20.0/ 16.3
LiOH (%-wt. of gelatin/ %-wt. of all non-water components)	0.8/ 0.8	1.1/ 1.0	1.2/ 1.2	1.7/ 1.5	2.5/ 2.0

TABLE S14.

Mixing of binder compositions comprising GA120 modified with TC in the presence of $Ca(OH)_2$ at pH 9

Mixing details about binder compositions comprising GA120 modified with TC in the presence of $Ca(OH)_2$ at pH 9 have been partly disclosed previously.[S2] The complete mixing details are listed in the Table S15. The binder compositions (all containing precipitates) were mixed using procedures analogous to the representative examples described in the article, using a stirred suspension of $Ca(OH)_2$ in water corresponding to 0.5 M $Ca(OH)_2$ both for pH adjustment and for mixing of the TC mixture (deep brown, paste/thick suspension, pH 8.0).

**Entry**	**1**	**2**	**3**	**4**	**5**
**Binder composition**					
GA120	100	100	100	100	100
TC (%-wt. of gelatin)	0	3	5	10	20
**Mixing of binder composition**					
GA120 (g)	12.00	12.00	12.00	12.00	11.00
Water (g)	64.43	65.42	65.92	66.95	62.83
0.5M $Ca(OH)_2$ (g)	4.40	4.86	5.31	6.00	8.17
pH	9.2	9.4	9.6	10.1	10.7
TC mixture (g)	0	1.62	2.70	5.40	9.90
pH	-	9.2	9.2	9.1	9.1
**Calculated binder composition**					
Binder concentration (%-wt. in water)	15.0	15.0	15.0	15.0	15.0
GA120 (%-wt. of gelatin/	100.0/	100.0/	100.0/	100.0/	100.0/
%-wt. of all non-water components)	98.7	95.4	93.3	88.5	79.9
TC (%-wt. of gelatin/	0/	3.0/	5.0/	10.0/	20.0/
%-wt. of all non-water components)	0	2.9	4.7	8.8	16.0
$Ca(OH)_2$ (%-wt. of gelatin/	1.3/	1.8/	2.2/	3.0/	5.2/
%-wt. of all non-water components)	1.3	1.7	2.1	2.7	4.1

TABLE S15.

Mixing of binder compositions comprising GA120 modified with TC in the presence of NaOH at pH 7

The mixing details of binder compositions comprising GA120 modified with TC in the presence of NaOH at pH 7 are listed in Table S16. The binder compositions were mixed using procedures analogous to the representative examples described in the article, using 0.5 M NaOH for mixing of the TC mixture (deep red-brown, pH 7.2) and adjusting the final pH to 7 with 1.0 M NaOH.

**Entry**	**1**	**2**	**3**	**4**	**5**
**Binder composition**					
GA120	100	100	100	100	100
TC (%-wt. of gelatin)	0	3	5	10	20
**Mixing of binder composition**					
GA120 (g)	12.00	12.00	12.00	12.00	11.00
Water (g)	65.42	66.43	66.95	69.08	66.46
1M NaOH (g)	3.10	3.10	3.10	3.35	3.30
pH	7.1	7.0	7.0	7.1	7.2
TC mixture (g)	0	1.62	2.70	5.40	9.90
pH	-	6.9	6.9	6.9	6.9
**Calculated binder composition**					
Binder concentration (%-wt. in water)	15.1	15.0	15.1	14.9	14.9
GA120 (%-wt. of gelatin/ %-wt. of all non-water components)	100.0/ 99.0	100.0/ 96.0	100.0/ 94.0	100.0/ 89.5	100.0/ 81.6
TC (%-wt. of gelatin/ %-wt. of all non-water components)	0/ 0	3.0/ 2.9	5.0/ 4.7	10.0/ 8.9	20.0/ 16.3
NaOH (%-wt. of gelatin/ %-wt. of all non-water components)	1.0/ 1.0	1.2/ 1.2	1.3/ 1.3	1.8/ 1.6	2.5/ 2.1

TABLE S16.

Mixing of binder compositions comprising GA120 modified with TC in the presence of NaOH at pH 8

The mixing details of binder compositions comprising GA120 modified with TC in the presence of NaOH at pH 8 are listed in Table S17. The binder compositions were mixed using procedures analogous to the representative examples described in the article, using 0.7 M NaOH for mixing of the TC mixture (deep red-brown, pH 8.0) and adjusting the final pH to 8 with 1.0 M NaOH.

**Entry**	**1**	**2**	**3**	**4**	**5**
**Binder composition**					
GA120	100	100	100	100	100
TC (%-wt. of gelatin)	0	3	5	10	20
**Mixing of binder composition**					
GA120 (g)	12.00	12.00	12.00	12.00	11.00
Water (g)	64.92	65.92	66.43	68.00	65.39
1M NaOH (g)	4.35	4.40	4.40	4.70	5.15
pH	8.2	8.1	8.2	8.3	8.4
TC mixture (g)	0	1.62	2.70	5.40	9.90
pH	-	7.9	7.9	7.9	8.0
**Calculated binder composition**					
Binder concentration (%-wt. in water)	15.0	15.0	15.0	15.0	14.9
GA120 (%-wt. of gelatin/ %-wt. of all non-water components)	100.0/ 98.6	100.0/ 95.5	100.0/ 93.6	100.0/ 88.9	100.0/ 80.8
TC (%-wt. of gelatin/ %-wt. of all non-water components)	0/ 0	3.0/ 2.9	5.0/ 4.7	10.0/ 8.9	20.0/ 16.2
NaOH (%-wt. of gelatin/ %-wt. of all non-water components)	1.4/ 1.4	1.7/ 1.6	1.9/ 1.8	2.5/ 2.2	3.7/ 3.0

TABLE S17.

Mixing of binder compositions comprising GA120 modified with TC in the presence of NaOH at pH 11

The mixing details of binder compositions comprising GA120 modified with TC in the presence of NaOH at pH 11 are listed in Table S18. The binder compositions were mixed using procedures analogous to the representative examples described in the article, using 1.5 M NaOH for mixing of the TC mixture (deep red-brown, pH 10.9) and adjusting the final pH to 11 with 1.0 M NaOH.

**Entry**	**1**	**2**	**3**	**4**	**5**
**Binder composition**					
GA120	100	100	100	100	100
TC (%-wt. of gelatin)	0	3	5	10	20
**Mixing of binder composition**					
GA120 (g)	12.00	12.00	12.00	12.00	11.00
Water (g)	60.29	61.62	62.53	64.43	63.32
1M NaOH (g)	10.10	10.00	10.50	10.90	11.20
pH	11.1	11.0	11.0	11.1	11.3
TC mixture (g)	0	1.62	2.70	5.40	9.90
pH	-	10.9	10.9	10.9	10.8
**Calculated binder composition**					
Binder concentration (%-wt. in water)	15.0	15.0	15.0	15.0	14.8
GA120 (%-wt. of gelatin/ %-wt. of all non-water components)	100.0/ 96.9	100.0/ 93.6	100.0/ 91.4	100.0/ 86.5	100.0/ 78.1
TC (%-wt. of gelatin/ %-wt. of all non-water components)	0/ 0	3.0/ 2.8	5.0/ 4.6	10.0/ 8.7	20.0/ 15.6
NaOH (%-wt. of gelatin/ %-wt. of all non-water components)	3.2/ 3.1	3.8/ 3.6	4.4/ 4.0	5.6/ 4.8	8.0/ 6.3

TABLE S18.

FURTHER DETAILED RESULTS FOR COMPOSITE BARS

Results for binder compositions comprising GA78 modified with TC in the presence of NaOH at pH 9

Details about mechanical strengths and binder contents for binder compositions comprising GA78 modified with TC in the presence of NaOH at pH 9 have been published previously.[S1] The water uptake results for binder compositions comprising GA78 modified with TC in the presence of NaOH at pH 9 are shown in Table S19 and Figure S4.

Entry	1	2	3	4	5	6	7
**Binder composition**							
GA78	100	100	100	100	100	100	100
TC (%-wt. of gelatin)	0	3	5	10	20	30	50
**Composite bar results**							
Avg. water uptake, 3 h (%, n = 3)	7.1	12.3	13.7	17.9	21.2	22.5	23.8
Standard error (%)	0.3	0.8	0.7	1.7	2.0	1.6	1.6
Avg. water uptake, 24 h (%, n = 3)	12.4	24.7	27.8	31.7	37.4	38.4	45.2
Standard error (%)	0.1	1.6	1.7	1.6	2.3	0.5	0.8

TABLE S19.

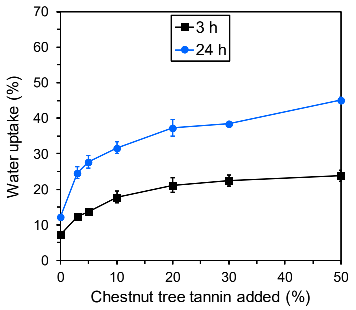

FIGURE S4.

Results for binder compositions comprising GA120 modified with TC in the presence of NaOH at pH 9

Details about mechanical strengths and binder contents for binder compositions comprising GA120 modified with TC in the presence of NaOH at pH 9 have been published previously.[S1] The water uptake results for binder compositions comprising GA120 modified with TC in the presence of NaOH at pH 9 are shown in Table S20 and Figure S5.

**Entry**	**1**	**2**	**3**	**4**	**5**	**6**	**7**
**Binder composition**							
GA120	100	100	100	100	100	100	100
TC (%-wt. of gelatin)	0	3	5	10	20	30	50
**Composite bar results**							
Avg. water uptake, 3 h (%, n = 3)	5.8	11.9	15.3	18.0	18.4	19.2	20.3
Standard error (%)	0.1	0.5	0.8	0.9	1.8	0.2	1.0
Avg. water uptake, 24 h (%, n = 3)	11.2	26.0	26.8	32.7	33.0	35.8	38.1
Standard error (%)	0.1	1.0	0.7	1.5	1.1	0.5	0.7

TABLE S20.

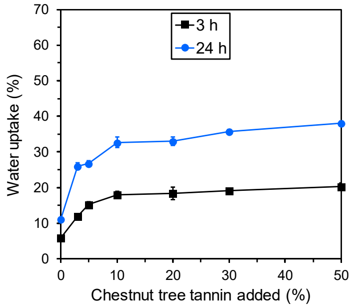

FIGURE S5.

Results for binder compositions comprising GA180 modified with TC in the presence of NaOH at pH 9

Details about mechanical strengths and binder contents for binder compositions comprising GA180 modified with TC in the presence of NaOH at pH 9 have been published previously.[S1] The water uptake results for binder compositions comprising GA180 modified with TC in the presence of NaOH at pH 9 are shown in Table S21 and Figure S6.

Entry	1	2	3	4	5	6	7
**Binder composition**							
GA180	100	100	100	100	100	100	100
TC (%-wt. of gelatin)	0	3	5	10	20	30	50
**Composite bar results**							
Avg. water uptake, 3 h (%, n = 3)	5.6	9.4	10.1	12.5	13.7	13.0	16.0
Standard error (%)	0.2	0.3	0.9	0.6	0.4	0.4	1.0
Avg. water uptake, 24 h (%, n = 3)	10.4	20.4	20.7	25.6	30.7	29.5	33.5
Standard error (%)	0.2	0.3	0.6	1.0	1.0	2.2	1.6

TABLE S21.

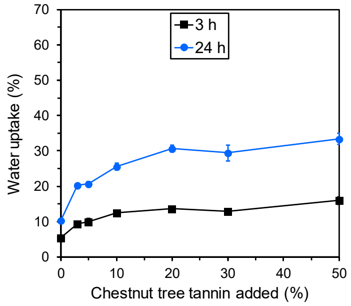

FIGURE S6.

Results for binder compositions comprising GA305 modified with TC in the presence of NaOH at pH 9

Details about mechanical strengths and binder contents for binder compositions comprising GA305 modified with TC in the presence of NaOH at pH 9 have been published previously.[S1] The water uptake results for binder compositions comprising GA305 modified with TC in the presence of NaOH at pH 9 are shown in Table S22 and Figure S7.

**Entry**	**1**	**2**	**3**	**4**	**5**	**6**	**7**
**Binder composition**							
GA305	100	100	100	100	100	100	100
TC (%-wt. of gelatin)	0	3	5	10	20	30	50
**Composite bar results**							
Avg. water uptake, 3 h (%, n = 3)	5.1	7.0	8.5	10.1	11.6	13.2	16.0
Standard error (%)	0.3	0.6	0.3	0.1	0.3	0.4	0.8
Avg. water uptake, 24 h (%, n = 3)	9.5	14.1	15.2	21.2	23.5	26.7	31.9
Standard error (%)	0.3	1.0	0.1	0.7	0.1	0.6	1.9

TABLE S22.

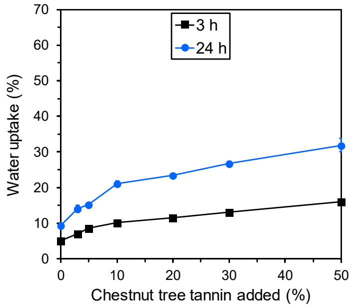

FIGURE S7.

Results for binder compositions comprising GA291v modified with TC in the presence of NaOH at pH 9

The results obtained for binder compositions comprising GA291v modified with TC in the presence of NaOH at pH 9 are shown in Table S23 and Figure S8.

**Entry**	**1**	**2**	**3**	**4**	**5**	**6**	**7**
**Binder composition**							
GA291v	100	100	100	100	100	100	100
TC (%-wt. of gelatin)	0	3	5	10	20	30	50
**Composite bar results**							
Avg. unaged mechanical strength (kN, n = 5)	0.251	0.303	0.322	0.302	0.273	0.226	0.148
Standard error (kN)	0.013	0.017	0.012	0.015	0.015	0.006	0.009
Binder content (%-wt of stone shots)	2.90	2.93	2.94	2.81	2.79	2.63	2.67
Avg. autoclave aged mech. strength (kN, n = 5)	0.252	0.289	0.241	0.216	0.192	0.172	0.129
Standard error (kN)	0.004	0.016	0.024	0.007	0.018	0.015	0.007
Binder content (%-wt of stone shots)	2.90	2.86	2.88	2.85	2.87	2.68	2.68
Avg. water bath aged mech. strength (kN, n = 5)	0	0.309	0.312	0.292	0.258	0.200	0.109
Standard error (kN)	-	0.024	0.017	0.010	0.011	0.009	0.006
Binder content (%-wt of stone shots)	0	2.76	2.80	2.79	2.70	2.56	2.45
Avg. water uptake, 3 h (%, n = 3)	5.9	7.6	8.1	10.4	13.5	14.4	16.3
Standard error (%)	0.3	0.5	0.3	0.3	0.8	0.5	0.7
Avg. water uptake, 24 h (%, n = 3)	10.3	18.3	18.6	23.1	29.7	32.2	33.3
Standard error (%)	0.1	1.3	0.2	0.8	1.3	0.6	0.5

TABLE S23.

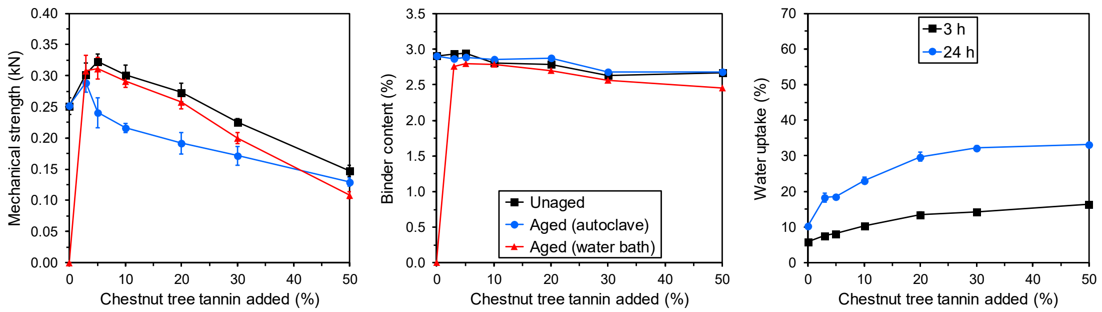

FIGURE S8.

Results for binder compositions comprising GB122 modified with TC in the presence of NaOH at pH 9

The results obtained for binder compositions comprising GB122 modified with TC in the presence of NaOH at pH 9 are shown in Table S24 and Figure S9.

Entry	1	2	3	4	5	6	7
**Binder composition**							
GB122	100	100	100	100	100	100	100
TC (%-wt. of gelatin)	0	3	5	10	20	30	50
**Composite bar results**							
Avg. unaged mechanical strength (kN, n = 5)	0.234	0.246	0.258	0.246	0.170	0.143	0.088
Standard error (kN)	0.018	0.006	0.013	0.016	0.012	0.012	0.009
Binder content (%-wt of stone shots)	2.81	2.79	2.80	2.76	2.71	2.69	2.63
Avg. autoclave aged mech. strength (kN, n = 5)	0.169	0.230	0.239	0.236	0.150	0.131	0.083
Standard error (kN)	0.006	0.022	0.022	0.017	0.009	0.013	0.008
Binder content (%-wt of stone shots)	2.80	2.77	2.77	2.72	2.67	2.63	2.57
Avg. water bath aged mech. strength (kN, n = 5)	0	0.139	0.154	0.140	0.134	0.091	0.057
Standard error (kN)	-	0.012	0.008	0.006	0.010	0.006	0.005
Binder content (%-wt of stone shots)	0	2.21	2.35	2.47	2.48	2.41	2.22
Avg. water uptake, 3 h (%, n = 3)	24.7	24.8	24.2	26.3	28.2	26.2	29.6
Standard error (%)	1.7	2.1	1.1	0.2	1.5	2.2	0.5
Avg. water uptake, 24 h (%, n = 3)	38.1	41.9	41.1	45.5	54.7	53.2	61.0
Standard error (%)	1.4	2.6	1.1	0.5	1.9	3.2	2.8

TABLE S24.

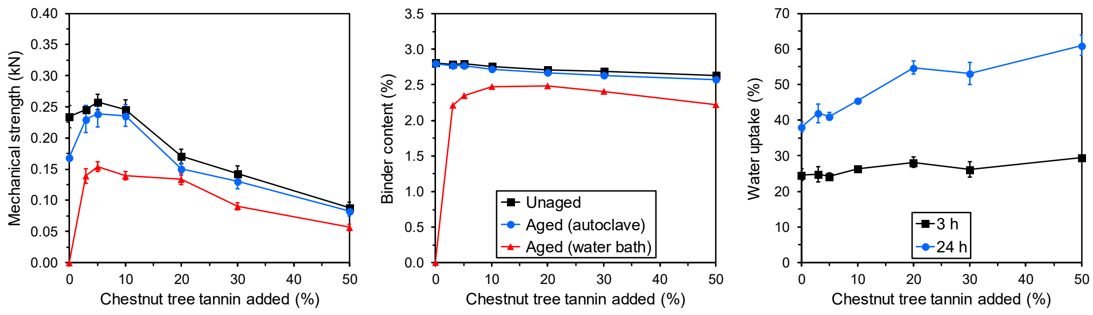

FIGURE S9.

Results for binder compositions comprising GB267 modified with TC in the presence of NaOH at pH 9

The results obtained for binder compositions comprising GB267 modified with TC in the presence of NaOH at pH 9 are shown in Table S25 and Figure S10.

**Entry**	**1**	**2**	**3**	**4**	**5**	**6**	**7**
**Binder composition**							
GB267	100	100	100	100	100	100	100
TC (%-wt. of gelatin)	0	3	5	10	20	30	50
**Composite bar results**							
Avg. unaged mechanical strength (kN, n = 5)	0.322	0.364	0.357	0.315	0.297	0.279	0.272
Standard error (kN)	0.014	0.08	0.015	0.019	0.012	0.011	0.017
Binder content (%-wt of stone shots)	3.01	3.00	2.93	2.94	2.92	2.85	2.83
Avg. autoclave aged mech. strength (kN, n = 5)	0.380	0.306	0.301	0.237	0.206	0.213	0.178
Standard error (kN)	0.012	0.016	0.020	0.012	0.011	0.019	0.011
Binder content (%-wt of stone shots)	2.97	2.96	2.89	2.86	2.78	2.77	2.71
Avg. water bath aged mech. strength (kN, n = 5)	0	0.192	0.212	0.201	0.151	0.161	0.112
Standard error (kN)	-	0.020	0.020	0.011	0.010	0.009	0.003
Binder content (%-wt of stone shots)	0	2.55	2.74	2.80	2.72	2.68	2.48
Avg. water uptake, 3 h (%, n = 3)	16.9	17.0	17.7	17.2	17.2	17.5	21.1
Standard error (%)	1.1	1.2	1.3	0.8	0.9	0.9	0.2
Avg. water uptake, 24 h (%, n = 3)	36.1	29.1	29.0	29.4	31.7	33.0	38.4
Standard error (%)	1.4	1.7	0.4	1.2	0.6	1.3	1.1

TABLE S25.

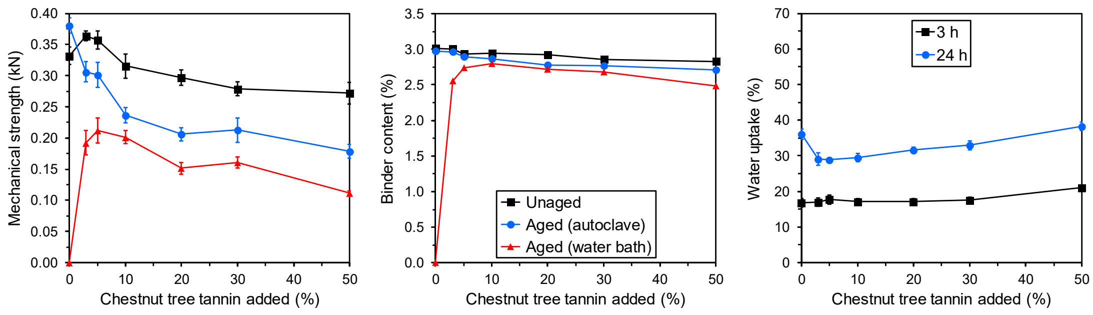

FIGURE S10.

Results for binder compositions comprising GB278vs modified with TC in the presence of NaOH at pH 9

The results obtained for binder compositions comprising GB278vs modified with TC in the presence of NaOH at pH 9 are shown in Table S26 and Figure S11.

**Entry**	**1**	**2**	**3**	**4**	**5**	**6**	**7**
**Binder composition**							
GB278vs	100	100	100	100	100	100	100
TC (%-wt. of gelatin)	0	3	5	10	20	30	50
**Composite bar results**							
Avg. unaged mechanical strength (kN, n = 5)	0.294	0.332	0.307	0.318	0.272	0.256	0.169
Standard error (kN)	0.010	0.022	0.022	0.014	0.016	0.008	0.012
Binder content (%-wt of stone shots)	2.85	2.82	2.85	2.75	2.70	2.65	2.68
Avg. autoclave aged mech. strength (kN, n = 5)	0.291	0.369	0.346	0.319	0.239	0.202	0.134
Standard error (kN)	0.005	0.023	0.016	0.022	0.023	0.014	0.012
Binder content (%-wt of stone shots)	2.80	2.79	2.76	2.72	2.69	2.66	2.65
Avg. water bath aged mech. strength (kN, n = 5)	0	0.301	0.302	0.257	0.213	0.197	0.129
Standard error (kN)	-	0.014	0.016	0.006	0.013	0.010	0.009
Binder content (%-wt of stone shots)	0	2.71	2.69	2.72	2.60	2.59	2.43
Avg. water uptake, 3 h (%, n = 3)	23.9	17.8	13.8	15.7	16.4	15.8	17.3
Standard error (%)	0.3	1.2	0.9	1.2	1.3	0.4	0.3
Avg. water uptake, 24 h (%, n = 3)	31.6	29.6	24.0	27.9	32.0	31.9	33.7
Standard error (%)	2.3	0.6	0.7	0.6	2.0	0.6	0.5

TABLE S26.

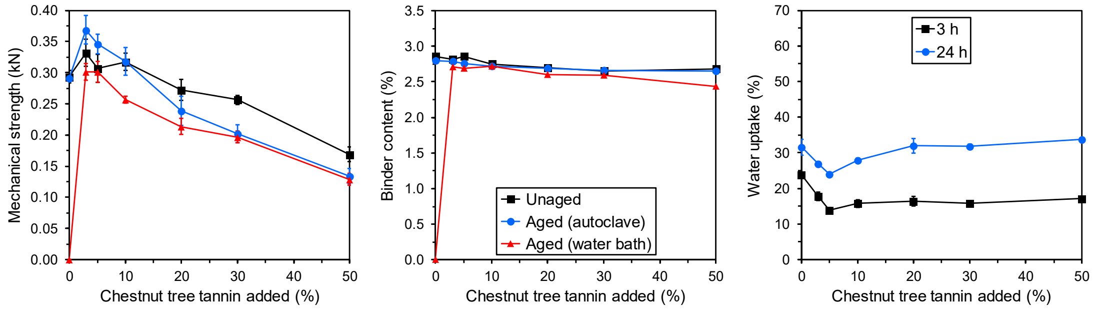

FIGURE S11.

Results for binder compositions comprising GA120/GA305 90:10 modified with TC in the presence of NaOH at pH 9

The results obtained for binder compositions comprising GA120/GA305 90:10 modified with TC in the presence of NaOH at pH 9 are shown in the Table S27 and Figure S12.

**Entry**	**1**	**2**	**3**	**4**	**5**
**Binder composition**					
GA120 + GA305	100	100	100	100	100
GA120 (%-wt. of gelatin)	90	90	90	90	90
GA305 (%-wt. of gelatin)	10	10	10	10	10
TC (%-wt. of gelatin)	0	3	5	10	20
**Composite bar results**					
Avg. unaged mechanical strength (kN, n = 5)	0.229	0.258	0.248	0.235	0.210
Standard error (kN)	0.009	0.014	0.013	0.011	0.011
Binder content (%-wt of stone shots)	2.78	2.80	2.77	2.77	2.75
Avg. autoclave aged mechanical strength (kN, n = 5)	0.223	0.222	0.225	0.218	0.192
Standard error (kN)	0.020	0.021	0.007	0.009	0.013
Binder content (%-wt of stone shots)	2.77	2.77	2.75	2.75	2.71
Avg. water bath aged mechanical strength (kN, n = 5)	0	0.143	0.176	0.174	0.125
Standard error (kN)	-	0.008	0.010	0.018	0.011
Binder content (%-wt of stone shots)	0	2.48	2.54	2.59	2.54
Avg. water uptake, 3 h (%, n = 3)	7.8	13.7	13.3	15.6	17.4
Standard error (%)	0.6	0.5	0.2	1.2	0.6
Avg. water uptake, 24 h (%, n = 3)	16.6	27.6	25.0	28.6	34.2
Standard error (%)	0.8	2.3	0.4	0.8	0.8

TABLE S27.

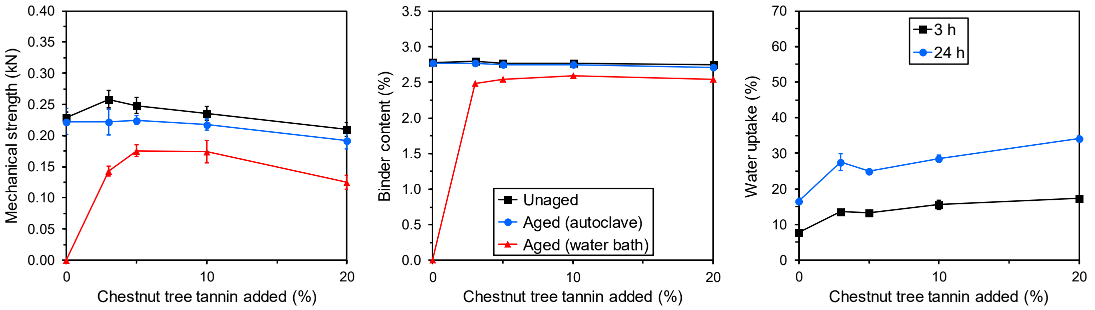

FIGURE S12.

Results for binder compositions comprising GA120/GB278vs 90:10 modified with TC in the presence of NaOH at pH 9

The results obtained for binder compositions comprising GA120/GB278vs 90:10 modified with TC in the presence of NaOH at pH 9 are shown in Table S28 and Figure S13.

**Entry**	**1**	**2**	**3**	**4**	**5**
**Binder composition**					
GA120 + GB278vs	100	100	100	100	100
GA120 (%-wt. of gelatin)	90	90	90	90	90
GB278vs (%-wt. of gelatin)	10	10	10	10	10
TC (%-wt. of gelatin)	0	3	5	10	20
**Composite bar results**					
Avg. unaged mechanical strength (kN, n = 5)	0.200	0.222	0.225	0.196	0.175
Standard error (kN)	0.018	0.021	0.014	0.016	0.016
Binder content (%-wt of stone shots)	2.76	2.78	2.77	2.74	2.74
Avg. autoclave aged mechanical strength (kN, n = 5)	0.180	0.206	0.216	0.201	0.176
Standard error (kN)	0.008	0.021	0.017	0.016	0.013
Binder content (%-wt of stone shots)	2.77	2.74	2.73	2.72	2.68
Avg. water bath aged mechanical strength (kN, n = 5)	0	0.191	0.208	0.219	0.205
Standard error (kN)	-	0.005	0.015	0.013	0.010
Binder content (%-wt of stone shots)	0	2.46	2.56	2.61	2.58
Avg. water uptake, 3 h (%, n = 3)	8.4	17.3	18.2	19.0	19.6
Standard error (%)	0.4	1.4	1.5	1.4	1.3
Avg. water uptake, 24 h (%, n = 3)	22.0	31.0	30.9	32.1	36.6
Standard error (%)	0.8	3.2	1.2	2.3	1.3

TABLE S28.

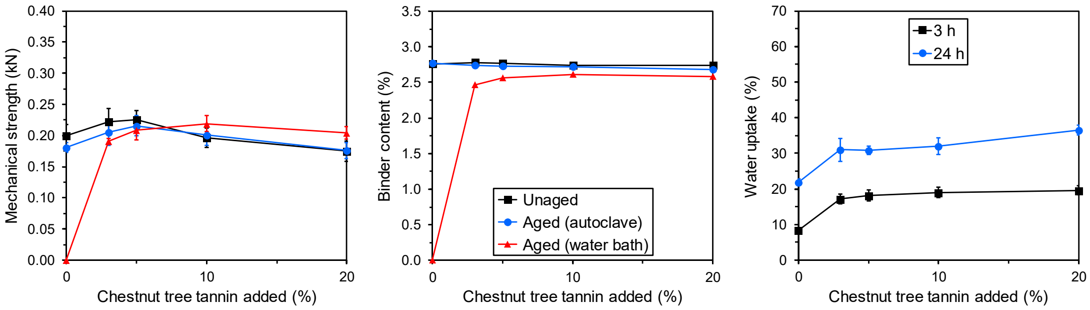

FIGURE S13.

Results for binder compositions comprising GA120 modified with TA in the presence of NaOH at pH 9

The results obtained for binder compositions comprising GA120 modified with TA in the presence of NaOH at pH 9 are shown in Table S29 and Figure S14.

Entry	1	2	3	4	5	6	7
**Binder composition**							
GA120	100	100	100	100	100	100	100
TA (%-wt. of gelatin)	0	3	5	10	20	30	50
**Composite bar results**							
Avg. unaged mechanical strength (kN, n = 5)	0.233	0.200	0.204	0.203	0.201	0.179	0.106
Standard error (kN)	0.017	0.008	0.007	0.013	0.018	0.004	0.003
Binder content (%-wt of stone shots)	2.77	2.75	2.72	2.71	2.61	2.57	2.51
Avg. autoclave aged mech. strength (kN, n = 5)	0.204	0.173	0.164	0.158	0.170	0.137	0.094
Standard error (kN)	0.019	0.017	0.010	0.016	0.008	0.004	0.005
Binder content (%-wt of stone shots)	2.75	2.72	2.69	2.68	2.59	2.52	2.42
Avg. water bath aged mech. strength (kN, n = 5)	0	0.122	0.131	0.112	0.091	0.053	0.012
Standard error (kN)	-	0.009	0.011	0.010	0.004	0.005	0.001
Binder content (%-wt of stone shots)	0	2.13	2.15	2.16	2.05	1.78	1.07
Avg. water uptake, 3 h (%, n = 3)	5.8	13.5	15.0	18.6	17.8	27.3	42.7
Standard error (%)	0.1	0.5	0.5	0.4	1.5	0.1	1.8
Avg. water uptake, 24 h (%, n = 3)	11.2	27.4	30.0	33.4	37.5	46.0	56.5
Standard error (%)	0.1	0.4	1.8	1.2	0.9	1.1	0.4

TABLE S29.

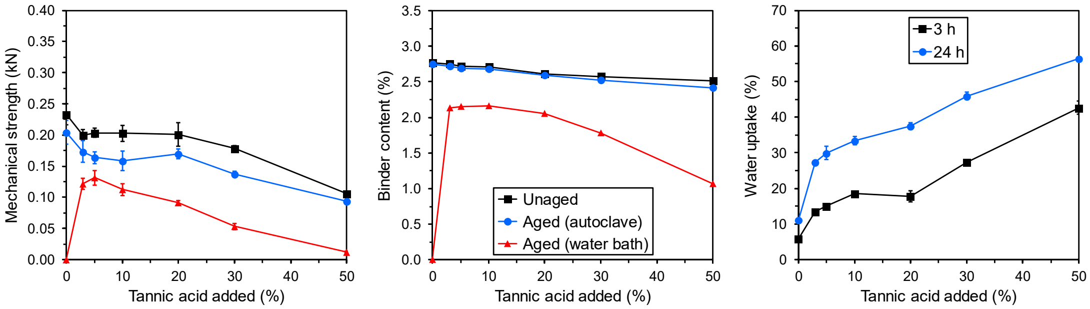

FIGURE S14.

Results for binder compositions comprising GA120 modified with TG in the presence of NaOH at pH 9

The results obtained for binder compositions comprising GA120 modified with TG in the presence of NaOH at pH 9 are shown in Table S30 and Figure S15.

Entry	1	2	3	4	5	6	7
**Binder composition**							
GA120	100	100	100	100	100	100	100
TG (%-wt. of gelatin)	0	3	5	10	20	30	50
**Composite bar results**							
Avg. unaged mechanical strength (kN, n = 5)	0.233	0.196	0.199	0.166	0.133	0.080	0.022
Standard error (kN)	0.017	0.009	0.013	0.016	0.006	0.004	0.001
Binder content (%-wt of stone shots)	2.77	2.72	2.71	2.71	2.69	2.67	2.70
Avg. autoclave aged mech. strength (kN, n = 5)	0.204	0.136	0.150	0.109	0.105	0.093	0.030
Standard error (kN)	0.019	0.008	0.011	0.009	0.007	0.008	0.003
Binder content (%-wt of stone shots)	2.75	2.68	2.67	2.67	2.64	2.64	2.68
Avg. water bath aged mech. strength (kN, n = 5)	0	0.089	0.015	0.102	0.071	0.051	0.010
Standard error (kN)	-	0.006	0.012	0.008	0.004	0.003	0.000
Binder content (%-wt of stone shots)	0	1.84	2.07	2.13	2.32	2.32	1.80
Avg. water uptake, 3 h (%, n = 3)	5.8	10.5	12.6	15.4	18.1	22.1	42.8
Standard error (%)	0.1	0.5	0.4	0.7	1.5	1.7	4.3
Avg. water uptake, 24 h (%, n = 3)	11.2	22.8	23.5	30.0	29.8	34.3	50.8
Standard error (%)	0.1	0.2	0.9	1.7	1.3	1.1	4.3

TABLE S30.

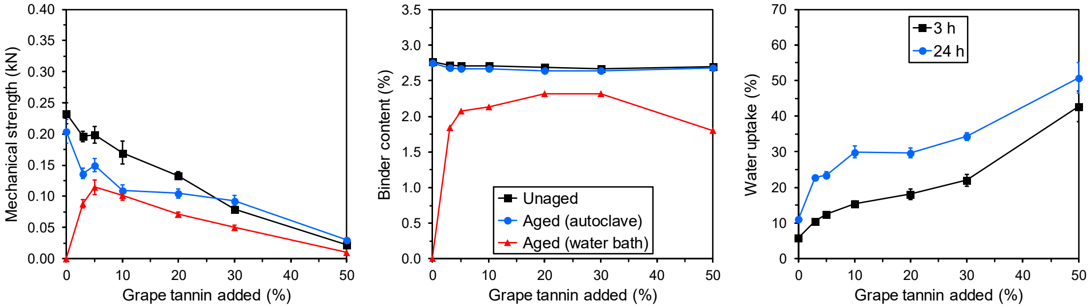

FIGURE S15.

Results for binder compositions comprising GA120 modified with TO in the presence of NaOH at pH 9

The results obtained for binder compositions comprising GA120 modified with TO in the presence of NaOH at pH 9 are shown in Table S31 and Figure S16.

Entry	1	2	3	4	5	6	7
**Binder composition**							
GA120	100	100	100	100	100	100	100
TO (%-wt. of gelatin)	0	3	5	10	20	30	50
**Composite bar results**							
Avg. unaged mechanical strength (kN, n = 5)	0.233	0.254	0.249	0.226	0.223	0.218	0.149
Standard error (kN)	0.017	0.008	0.010	0.018	0.005	0.015	0.013
Binder content (%-wt of stone shots)	2.77	2.82	2.78	2.77	2.74	2.73	2.69
Avg. autoclave aged mech. strength (kN, n = 5)	0.204	0.248	0.241	0.231	0.209	0.177	0.098
Standard error (kN)	0.019	0.022	0.024	0.019	0.016	0.013	0.004
Binder content (%-wt of stone shots)	2.75	2.78	2.76	2.73	2.71	2.67	2.63
Avg. water bath aged mech. strength (kN, n = 5)	0	0.185	0.211	0.180	0.184	0.131	0.064
Standard error (kN)	-	0.017	0.012	0.014	0.010	0.008	0.006
Binder content (%-wt of stone shots)	0	2.52	2.54	2.54	2.51	2.44	2.18
Avg. water uptake, 3 h (%, n = 3)	5.8	15.8	14.2	15.6	13.8	15.3	31.8
Standard error (%)	0.1	1.5	0.8	1.3	0.2	1.0	1.2
Avg. water uptake, 24 h (%, n = 3)	11.2	26.7	25.8	32.1	30.2	35.2	49.3
Standard error (%)	0.1	2.2	0.7	0.7	1.4	2.0	2.8

TABLE S31.

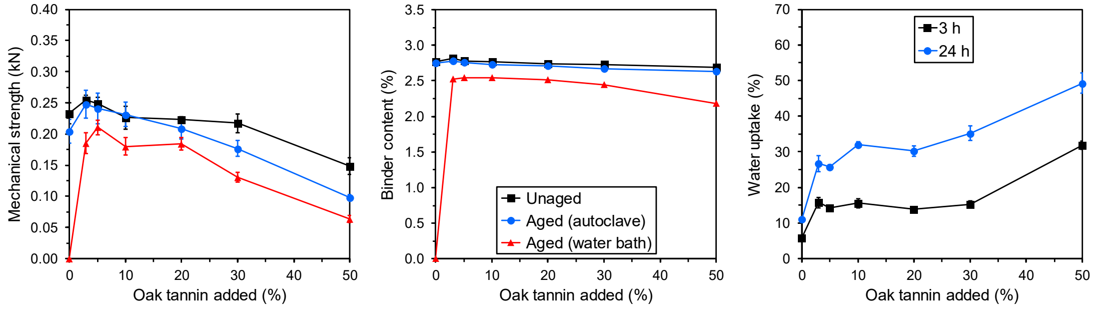

FIGURE S16.

Results for binder compositions comprising GA120 modified with TQ in the presence of NaOH at pH 9

The results obtained for binder compositions comprising GA120 modified with TQ in the presence of NaOH at pH 9 are shown in Table S32 and Figure S17.

Entry	1	2	3	4	5	6	7
**Binder composition**							
GA120	100	100	100	100	100	100	100
TQ (%-wt. of gelatin)	0	3	5	10	20	30	50
**Composite bar results**							
Avg. unaged mechanical strength (kN, n = 5)	0.233	0.211	0.245	0.239	0.222	0.149	0.066
Standard error (kN)	0.017	0.015	0.020	0.020	0.011	0.004	0.006
Binder content (%-wt of stone shots)	2.77	2.82	2.81	2.79	2.78	2.87	2.77
Avg. autoclave aged mech. strength (kN, n = 5)	0.204	0.218	0.213	0.189	0.176	0.118	0.058
Standard error (kN)	0.019	0.017	0.019	0.003	0.008	0.003	0.009
Binder content (%-wt of stone shots)	2.75	2.76	2.77	2.75	2.75	2.83	2.77
Avg. water bath aged mech. strength (kN, n = 5)	0	0.146	0.178	0.177	0.170	0.118	0.058
Standard error (kN)	-	0.007	0.012	0.010	0.030	0.010	0.005
Binder content (%-wt of stone shots)	0	2.24	2.38	2.45	2.53	2.66	2.48
Avg. water uptake, 3 h (%, n = 3)	5.8	11.9	11.9	15.4	14.1	13.2	16.2
Standard error (%)	0.1	1.1	0.5	0.2	0.9	0.3	1.1
Avg. water uptake, 24 h (%, n = 3)	11.2	30.6	28.0	27.3	27.5	27.5	31.8
Standard error (%)	0.1	2.4	1.8	1.3	0.8	1.9	0.9

TABLE S32.

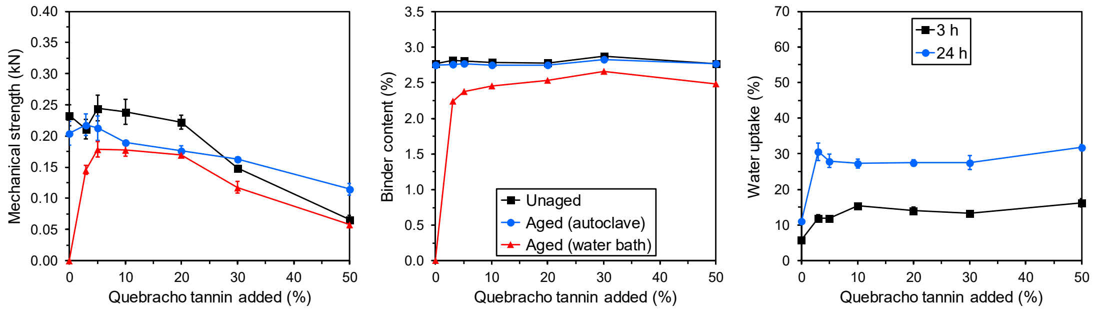

FIGURE S17.

Results for binder compositions comprising GA120 modified with TC in the presence of KOH at pH 9

The results obtained for binder compositions comprising GA120 modified with TC in the presence of KOH at pH 9 are shown in Table S33 and Figure S18.

**Entry**	**1**	**2**	**3**	**4**	**5**
**Binder composition**					
GA120	100	100	100	100	100
TC (%-wt. of gelatin)	0	3	5	10	20
**Composite bar results**					
Avg. unaged mechanical strength (kN, n = 5)	0.194	0.249	0.242	0.234	0.227
Standard error (kN)	0.019	0.018	0.014	0.004	0.010
Binder content (%-wt of stone shots)	2.89	2.90	2.79	2.83	2.78
Avg. autoclave aged mechanical strength (kN, n = 5)	0.182	0.177	0.191	0.189	0.174
Standard error (kN)	0.010	0.014	0.008	0.006	0.009
Binder content (%-wt of stone shots)	2.84	2.79	2.76	2.77	2.71
Avg. water bath aged mechanical strength (kN, n = 5)	0	0.148	0.165	0.184	0.171
Standard error (kN)	-	0.010	0.011	0.019	0.010
Binder content (%-wt of stone shots)	0	2.42	2.55	2.56	2.55
Avg. water uptake, 3 h (%, n = 3)	5.9	12.0	12.3	17.0	15.3
Standard error (%)	0.3	1.0	1.0	0.8	0.4
Avg. water uptake, 24 h (%, n = 3)	12.7	26.6	26.5	30.5	33.0
Standard error (%)	0.3	1.8	0.9	1.2	1.0

TABLE S33.

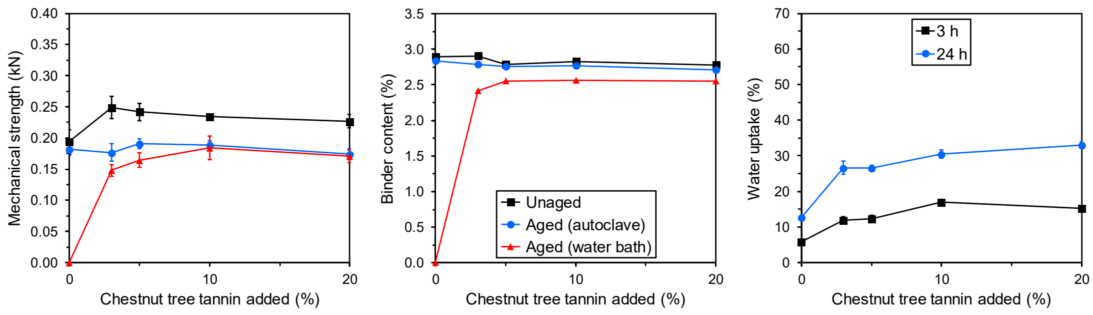

FIGURE S18.

Results for binder compositions comprising GA120 modified with TC in the presence of LiOH at pH 9

The results obtained for binder compositions comprising GA120 modified with TC in the presence of LiOH at pH 9 are shown in Table S34 and Figure S19.

**Entry**	**1**	**2**	**3**	**4**	**5**
**Binder composition**					
GA120	100	100	100	100	100
TC (%-wt. of gelatin)	0	3	5	10	20
**Composite bar results**					
Avg. unaged mechanical strength (kN, n = 5)	0.179	0.230	0.215	0.224	0.195
Standard error (kN)	0.008	0.023	0.013	0.009	0.004
Binder content (%-wt of stone shots)	2.94	2.96	2.95	2.95	2.91
Avg. autoclave aged mechanical strength (kN, n = 5)	0.193	0.203	0.201	0.192	0.174
Standard error (kN)	0.010	0.012	0.020	0.006	0.008
Binder content (%-wt of stone shots)	2.89	2.89	2.91	2.87	2.84
Avg. water bath aged mechanical strength (kN, n = 5)	0	0.175	0.187	0.178	0.161
Standard error (kN)	-	0.012	0.010	0.005	0.005
Binder content (%-wt of stone shots)	0	2.61	2.69	2.70	2.67
Avg. water uptake, 3 h (%, n = 3)	5.9	10.3	12.7	16.1	16.7
Standard error (%)	0.1	0.4	0.7	0.7	1.4
Avg. water uptake, 24 h (%, n = 3)	13.7	22.9	25.4	30.0	35.3
Standard error (%)	0.4	0.4	0.4	0.7	0.4

TABLE S34.

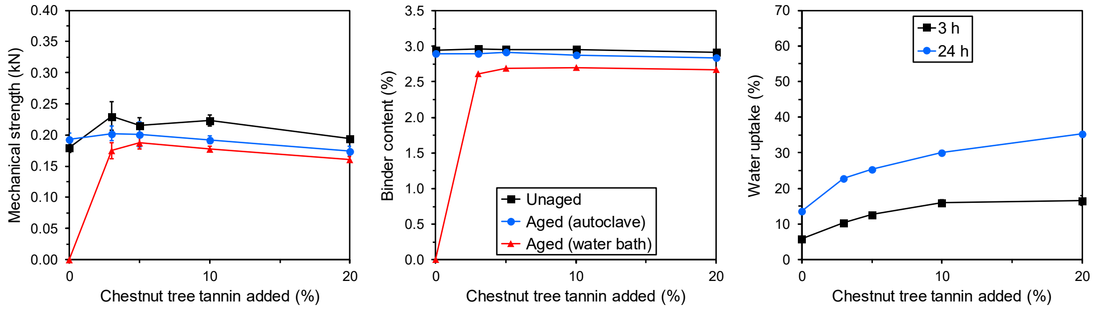

FIGURE S19.

Results for binder compositions comprising GA120 modified with TC in the presence of $Ca(OH)_2$ at pH 9

The results obtained for binder compositions comprising GA120 modified with TC in the presence of $Ca(OH)_2$ at pH 9 have been partly disclosed previously.[S2] The complete results obtained for binder compositions comprising GA120 modified with TC in the presence of $Ca(OH)_2$ at pH 9 are shown in Table S35 and Figure S20.

**Entry**	**1**	**2**	**3**	**4**	**5**
**Binder composition**					
GA120	100	100	100	100	100
TC (%-wt. of gelatin)	0	3	5	10	20
**Composite bar results**					
Avg. unaged mechanical strength (kN, n = 5)	0.198	0.220	0.232	0.222	0.210
Standard error (kN)	0.020	0.018	0.011	0.022	0.012
Binder content (%-wt of stone shots)	2.87	2.86	2.89	2.81	2.79
Avg. autoclave aged mechanical strength (kN, n = 5)	0.191	0.200	0.191	0.156	0.151
Standard error (kN)	0.014	0.015	0.017	0.008	0.012
Binder content (%-wt of stone shots)	2.83	2.80	2.82	2.73	2.71
Avg. water bath aged mechanical strength (kN, n = 5)	0	0	0	0.106	0.163
Standard error (kN)	-	-	-	0.007	0.013
Binder content (%-wt of stone shots)	0	0	0	1.89	2.23
Avg. water uptake, 3 h (%, n = 3)	5.5	5.8	5.3	5.4	5.0
Standard error (%)	0.2	0.4	0.4	0.2	0.2
Avg. water uptake, 24 h (%, n = 3)	10.8	10.3	10.0	10.3	10.4
Standard error (%)	0.3	0.5	0.5	0.2	0.4

TABLE S35.

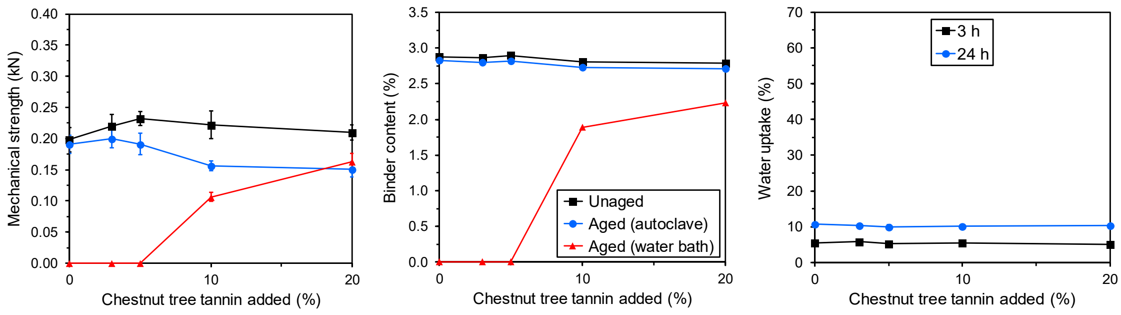

FIGURE S20.

Results for binder compositions comprising GA120 modified with TC in the presence of NaOH at pH 7

The results obtained for binder compositions comprising GA120 modified with TC in the presence of NaOH at pH 7 are shown in Table S36 and Figure S21.

**Entry**	**1**	**2**	**3**	**4**	**5**
**Binder composition**					
GA120	100	100	100	100	100
TC (%-wt. of gelatin)	0	3	5	10	20
**Composite bar results**					
Avg. unaged mechanical strength (kN, n = 5)	0.216	0.259	0.249	0.245	0.202
Standard error (kN)	0.013	0.006	0.010	0.012	0.010
Binder content (%-wt of stone shots)	2.80	2.79	2.81	2.76	2.74
Avg. autoclave aged mechanical strength (kN, n = 5)	0.213	0.227	0.252	0.224	0.179
Standard error (kN)	0.011	0.011	0.016	0.013	0.016
Binder content (%-wt of stone shots)	2.79	2.79	2.77	2.72	2.70
Avg. water bath aged mechanical strength (kN, n = 5)	0	0.064	0.091	0.095	0.066
Standard error (kN)	-	0.011	0.013	0.005	0.010
Binder content (%-wt of stone shots)	0	1.36	1.83	1.94	2.06
Avg. water uptake, 3 h (%, n = 3)	-	6.1	7.7	10.9	14.9
Standard error (%)	-	0.2	0.7	0.6	0.7
Avg. water uptake, 24 h (%, n = 3)	-	18.0	20.1	26.1	30.6
Standard error (%)	-	1.4	2.0	1.2	0.6

TABLE S36.

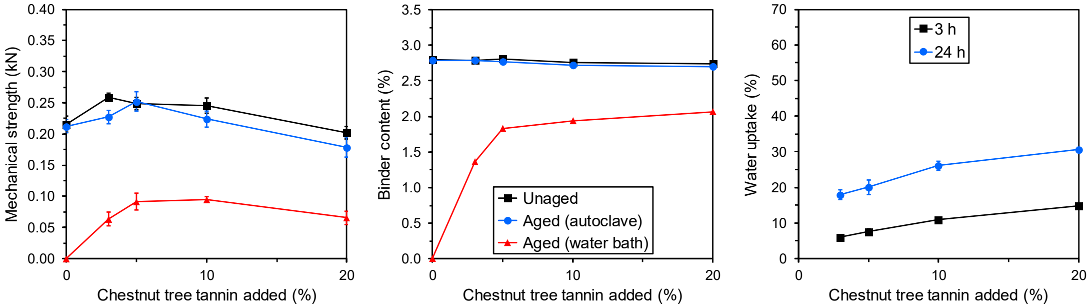

FIGURE S21.

Results for binder compositions comprising GA120 modified with TC in the presence of NaOH at pH 8

The results obtained for binder compositions comprising GA120 modified with TC in the presence of NaOH at pH 8 are shown in Table S37 and Figure S22.

**Entry**	**1**	**2**	**3**	**4**	**5**
**Binder composition**					
GA120	100	100	100	100	100
TC (%-wt. of gelatin)	0	3	5	10	20
**Composite bar results**					
Avg. unaged mechanical strength (kN, n = 5)	0.236	0.221	0.264	0.271	0.229
Standard error (kN)	0.025	0.022	0.014	0.013	0.008
Binder content (%-wt of stone shots)	2.84	2.86	2.80	2.80	2.73
Avg. autoclave aged mechanical strength (kN, n = 5)	0.227	0.203	0.231	0.242	0.220
Standard error (kN)	0.024	0.016	0.011	0.014	0.004
Binder content (%-wt of stone shots)	2.79	2.78	2.75	2.75	2.71
Avg. water bath aged mechanical strength (kN, n = 5)	0	0.093	0.145	0.166	0.140
Standard error (kN)	-	0.015	0.010	0.011	0.013
Binder content (%-wt of stone shots)	0	2.12	2.33	2.43	2.43
Avg. water uptake, 3 h (%, n = 3)	6.2	10.5	10.9	13.0	17.1
Standard error (%)	0.2	0.4	0.6	1.3	1.0
Avg. water uptake, 24 h (%, n = 3)	12.4	24.5	25.2	27.7	34.3
Standard error (%)	0.1	2.1	0.6	0.9	2.5

TABLE S37.

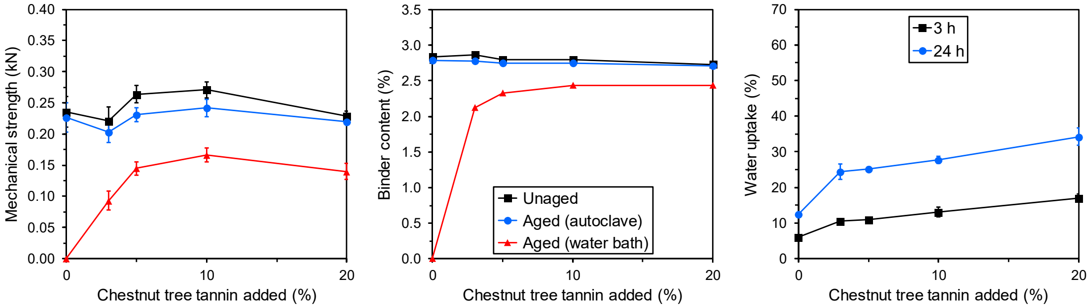

FIGURE S22.

Results for binder compositions comprising GA120 modified with TC in the presence of NaOH at pH 11

The results obtained for binder compositions comprising GA120 modified with TC in the presence of NaOH at pH 11 are shown in Table S38 and Figure S23.

**Entry**	**1**	**2**	**3**	**4**	**5**
**Binder composition**					
GA120	100	100	100	100	100
TC (%-wt. of gelatin)	0	3	5	10	20
**Composite bar results**					
Avg. unaged mechanical strength (kN, n = 5)	0.130	0.180	0.177	0.192	0.177
Standard error (kN)	0.004	0.005	0.014	0.018	0.012
Binder content (%-wt of stone shots)	2.84	2.84	2.82	2.76	2.69
Avg. autoclave aged mechanical strength (kN, n = 5)	0.071	0.116	0.128	0.151	0.123
Standard error (kN)	0.006	0.011	0.011	0.014	0.009
Binder content (%-wt of stone shots)	2.75	2.73	2.70	2.67	2.58
Avg. water bath aged mechanical strength (kN, n = 5)	0	0.149	0.177	0.185	0.136
Standard error (kN)	-	0.008	0.017	0.004	0.007
Binder content (%-wt of stone shots)	0	2.40	2.54	2.57	2.46
Avg. water uptake, 3 h (%, n = 3)	18.9	18.5	15.3	13.4	16.7
Standard error (%)	0.6	0.7	0.3	0.2	0.7
Avg. water uptake, 24 h (%, n = 3)	33.7	34.1	30.5	30.0	43.9
Standard error (%)	1.4	1.4	0.3	1.5	0.5

TABLE S38.

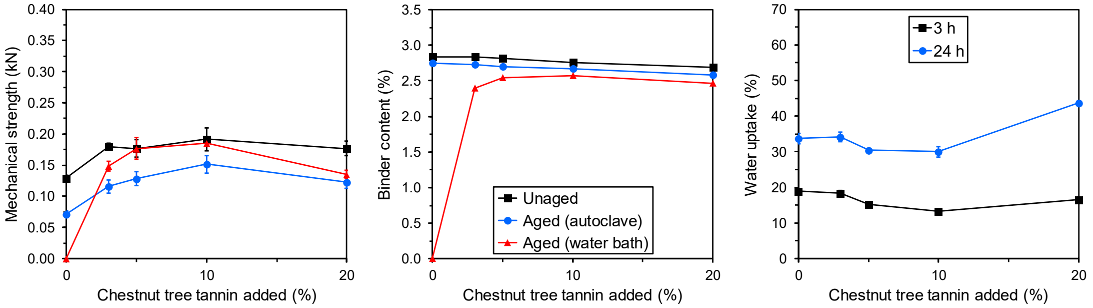

FIGURE S23.

REFERENCES

(S1) Hjelmgaard, T.; Thorsen, P. A.; Bøtner, J. A.; Kaurin, J.; Schmücker, C. M.; Nærum, L. Towards greener stone shot and stone wool materials: binder systems based on gelatine modified with tannin or transglutaminase. *Green. Chem.* **2018**, *20*, 4102-4111.

(S2) Hjelmgaard, T. *Mineral wool binder*; WO2021032645A1, 2021.
